# An overview of gene expression dynamics during early ovarian folliculogenesis: specificity of follicular compartments and bi-directional dialog

**DOI:** 10.1186/1471-2164-14-904

**Published:** 2013-12-19

**Authors:** Agnes Bonnet, Cedric Cabau, Olivier Bouchez, Julien Sarry, Nathalie Marsaud, Sylvain Foissac, Florent Woloszyn, Philippe Mulsant, Beatrice Mandon-Pepin

**Affiliations:** 1 INRA, UMR444 Génétique Cellulaire, Auzeville, BP52627, F-31326, Castanet-Tolosan, France; 2 ENVT, UMR444, Laboratoire de Génétique Cellulaire, F-31326 Castanet-Tolosan, France; 3 INRA, SIGENAE, UR83 Recherches Avicoles, F-37380 Nouzilly, France; 4 GeT-PlaGe, Genotoul, INRA Auzeville, F31326 Castanet-tolosan, France; 5 Université de Toulouse; INSA, UPS, INP; LISBP, 135 Avenue de Rangueil, F-31077 Toulouse, France; 6 INRA, UMR792, Ingénierie des Systèmes Biologiques et des Procédés, F-31400 Toulouse, France; 7 CNRS, UMR5504, F-31400 Toulouse, France; 8 INRA, UMR1198 Biologie du Développement et de la Reproduction, F-78350 Jouy-en-Josas, France

**Keywords:** RNA-seq, Early folliculogenesis, Molecular dialog, Transcriptome, Sheep, Oocyte, Granulosa cells, Microdissection

## Abstract

**Background:**

Successful early folliculogenesis is crucial for female reproductive function. It requires appropriate gene specific expression of the different types of ovarian cells at different developmental stages. To date, most gene expression studies on the ovary were conducted in rodents and did not distinguish the type of cell. In mono-ovulating species, few studies have addressed gene expression profiles and mainly concerned human oocytes.

**Results:**

We used a laser capture microdissection method combined with RNA-seq technology to explore the transcriptome in oocytes and granulosa cells (GCs) during development of the sheep ovarian follicle. We first documented the expression profile of 15 349 genes, then focused on the 5 129 genes showing differential expression between oocytes and GCs. Enriched functional categories such as oocyte meiotic arrest and GC steroid synthesis reflect two distinct cell fates. We identified the implication of GC signal transduction pathways such as SHH, WNT and RHO GTPase. In addition, signaling pathways (VEGF, NOTCH, IGF1, etc.) and GC transzonal projections suggest the existence of complex cell-cell interactions. Finally, we highlighted several transcription regulators and specifically expressed genes that likely play an important role in early folliculogenesis.

**Conclusions:**

To our knowledge, this is the first comprehensive exploration of transcriptomes derived from *in vivo* oocytes and GCs at *key* stages in early follicular development in sheep*.* Collectively, our data advance our understanding of early folliculogenesis in mono-ovulating species and will be a valuable resource for unraveling human ovarian dysfunction such as premature ovarian failure (POF).

## Background

The major function of an ovary is to produce oocytes intended for fertilization as eggs and to create a favorable environment for the beginning of gestation. In adult mammals, the ovary is a heterogeneous organ containing follicles at various stages of development and *corpora lutea,* either active or at various stages of involution. The formation of primordial follicles (oocytes surrounded by flattened pre-granulosa cells) occurs during fetal development in many species including human, sheep (from 75 days of gestation) [1], cattle, and goat, or after birth like in rodents. The primordial follicles represent a reserve of germ cells for the entire reproductive life of the female. They remain dormant until their recruitment and irreversible growth towards the primary, secondary and tertiary stages (with an antral cavity). The gradual exit of primordial follicles begins shortly after the formation of the primordial follicle pool and continues throughout the reproductive years [2]. Consequently, this early development is important as it regulates the size of the resting primordial follicle pool and the fate of the follicles, which, in turn, affects fertility and the reproductive life span.

Early follicular development requires the appropriate expression of many genes at different developmental stages. Recent studies on natural mutations in sheep [3] and on mutant mouse models demonstrated that the expression of different oocyte-specific genes is essential during early folliculogenesis in a stage specific expression pattern [2]. *Figla*, *Nobox*, *Pten*, *Foxo3A* and nerve growth factor genes are involved in the formation of primordial follicles, whereas genes such as *βFGF*, *GDF 9* and *BMP 4* are involved in the transition from primordial to primary follicles. Other genes such as *BMP15* are not expressed in the oocyte until the primary follicle stage and are involved in the transition from primary to secondary follicles, as shown in sheep (for a review see [4]).

This process also requires orchestrated communication between oocytes and granulosa cells (GCs), which are the structural components of the early follicle. Oocytes and GCs regulate follicle growth in an autocrine and paracrine manner via secreted factors and direct gap junctional communications. On one hand, it is assumed that oocytes control the proliferation of GCs and later, their differentiation into steroid secreting cells and their metabolism [5]. On the other hand, GCs are indispensable to oocyte growth, meiosis, cytoplasmic maturation and control of transcriptional activity within the oocyte [6]. This dialog also takes place through GC cytoplasmic extensions that penetrate the *zona pellucida* (ZP) and form gap junctions with oocyte cell membrane [7].

Although some large scale expression studies have been conducted on rodent and human oocytes [8-11], expression profiling of follicular compartments is difficult to accomplish due to the very small size of the follicles and the mixture of preantral stages in the ovary. Two studies in particular identified the specific transcriptome of human oocytes in the quiescent state [10,11]. These studies highlighted enriched functions (transcription, RNA post transcriptional modifications) and molecular mechanisms (cell cycle, androgen signaling) in human oocytes, which were also identified in the only preliminary analysis conducted in sheep species to date [12]. But exactly which granulosa cell factors play a specialized role in early follicular development is not known. Our knowledge of the key mechanisms and the bidirectional communication underlying early folliculogenesis is consequently still very limited and is mainly derived from rodents (poly-ovulating species). Spatial and temporal information on gene expression patterns would facilitate the identification of the regulatory gene networks that underlie cell growth, differentiation, and the functional specification of each follicular compartment.

The purpose of this study was thus to 1- describe global gene expression during early ovarian folliculogenesis in each follicular compartment (oocyte, GCs) in mono-ovulating species using sheep as the experimental model (whose fertility, litter size, ovulation rate and fetus development is similar to humans, unlike rodents); 2- identify differential and specific gene expression between these two follicular compartments; 3- investigate specific functions and pathways, and 4- explore bi-directional communication between oocytes and GCs. This was achieved using laser capture microdissection (LCM) to isolate oocytes and GCs during the earliest stages of folliculogenesis. Gene expression profiles were monitored at each follicle stage according to cell type using RNA-seq technology. We report the successful isolation and generation of global gene expression data sets of pure oocytes and GC populations at key stages of early follicular development*.* We describe the expression profile of 15 349 genes with 33.4% of genes displaying differential expression between the two compartments. In addition, we identified oocyte and GC-specific gene expression and the pathways involved in oocyte-GC communication.

## Results

We combined laser capture microdissection (LCM) [12] and RNA-sequencing technologies to detect separate GCs and oocyte global transcriptome for each stage of follicle development: primordial (PD), primary (PM), secondary (SC) follicles and the small antral stage (SA) as control. Three/four biological replicates were obtained per condition (8 conditions: cell type X stage). For the experimental protocol, see Additional file 1: Figure S1.

**Figure 1:**
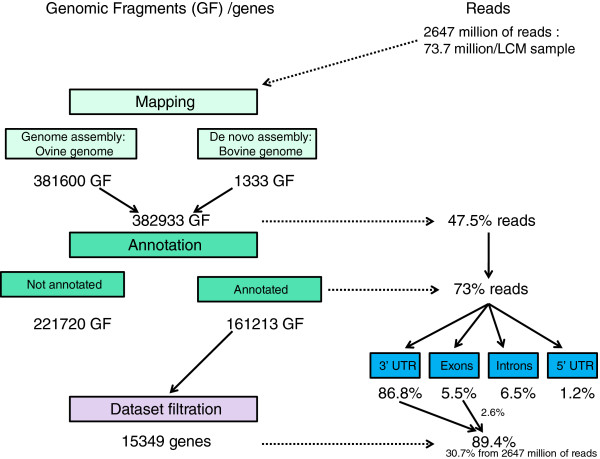
**Summary of bioinformatics processes.** The RNA-seq experiment produced a collection of 382,933 fragments that aggregated 47.5% of the LCM-aRNA reads. The annotation strategy assigned 73% of the mapped reads. A total of 221,716 genomic fragments remain unannotated. A total of 86.8% of the annotated reads are located in stop codon or 3′UTR regions, whereas only 5.5% are located in exons, 1.2% in start codon/ 5′UTR regions and 6.5% in introns. The final dataset conserved a single fragment per gene corresponding to the nearest 3′UTR region with the highest number of reads. This dataset aggregated 89.4% of the annotated LCM-aRNA reads (86.8% were located in 3′ UTR regions and 2.6% were located in exons).

The RNA-seq experiment generated around 2.647 billion 100 bp reads with an average of 73.7 million per LCM-derived amplified RNA sample (LCM-aRNA). The result of the bioinformatics and annotation processing is summarized in Figure 1 and detailed in Additional file 2. Briefly, the genome assembly approach (mapping to sheep genome sequence, assembly, followed by read counting) generated a collection of 382 933 fragments that aggregated 47.5% of LCM-aRNA reads. After 3′UTR selection, the final dataset included 15 349 genes. 221 716 genomic fragments remained unannotated.

### Exploring global gene expression

The 15 349 genes correspond to 62% of the bovine annotated genes in Ensembl UMD3.1 release 65, and to 78.6% of the ovine annotated genes in CSIRO Oarv2.0 (18 656 genes). As expected, the number of expressed genes was uniformly distributed between chromosomes according to the number of expected genes except for chromosome 15 on which significantly fewer genes were expressed (Additional file 1: Figure S2A). The percentage of genes transcribed for this chromosome was 57%, compared to an average of 80% for the other chromosomes (SD = 7.9%). The chromosome coverage of the expressed genes overlays Oarv2.0 annotated genes (Additional file 1: Figure S2B) except for chromosome 15 on which we identified two regions with fewer expressed genes. The density of expressed genes in these two regions (45–50 MB and 77–80 Mb) was 75%-90% lower.

For each sample, the expression levels were measured in read counts per gene (the 3′ UTR fragment being the most representative) and a normalized expression (RPM = sample gene read count/(sample total mapped read /1 000 000)) was calculated to describe the transcriptome of the tissue. First, we visualized a dynamic range of gene expression between 0.2 and 1 000 RPM (Additional file 1: Figure S3A-B). We estimated that 95% of the genes aggregated 56% of the reads. We then examined the highest expressed genes and detected a clear distinction between cell types (Additional file 1: Figures S3C-D). The highest oocyte-expressed genes contributed less to the total number of reads than GCs and multi-tissue samples (MT: 12 different tissues). For example, the ten highest oocyte-expressed genes contributed 4.2% to 4.7% of the total reads, while the highest expressed GC and MT genes contributed respectively 5% to 9.2% and 16.84% of the total reads. In oocytes, the most expressed genes included *ZP2*, *ZP3* and *GDF9*, genes known to be oocyte specific, but also *ACTG*, *PAIP1*, *PDK3*. In GCs, the most expressed genes included mainly ribosomal genes, such as *RPL4*, *RPL14*, *RPS23,* genes involved in cellular assembly and organization, such as *ACTG*, collagens, *VIM* and cell growth regulators, such as *MGP*.

**Figure 2:**
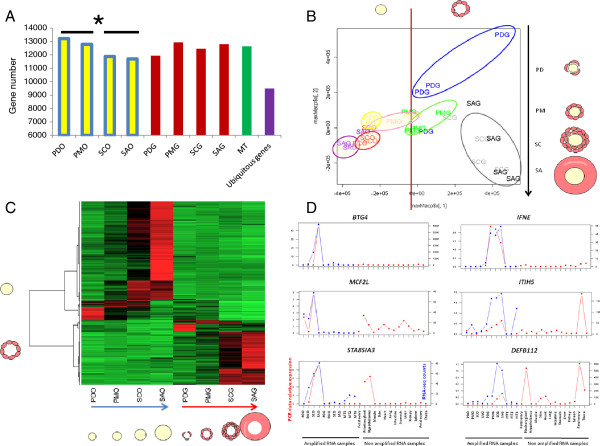
**Global gene expression. A**: Number of expressed genes. For each condition (stage x compartment) expressed genes are genes with an average expression >10 reads for ¾ of the replicates. Ubiquitous genes are genes expressed in all the samples. *: pval < 0.05 between PDO-PMO and SCO-SAO. **B**: Principal component analysis (PCA) of the transcriptome. PCA was performed on the gene data set after normalization using the R DEseq package. The first axis explains 52% of the expression variability and separates the two follicular compartments (O vs. GCs). The second axis explains 15% of the expression variability and separates the samples according to their follicular stages. **C**: Heatmap display of supervised hierarchical clustering of all the differential genes between compartments. The 1,694 genes are displayed in rows and the mean of replicates per condition are displayed in columns. Red, black and green represent up, mean, and down expression, respectively. **D**: Specific expression profiles. Relative quantification of *BTG4 MCF2L, STA8SIA3* oocyte-specific genes and *IFNE, ITIH5, DEFB112* GC-specific genes in the 2 follicular compartments and throughout early follicular development ((n = 3-4), Pd: primordial, Pm: primary, Sc: secondary, SA: small antrum). Expression specificity was checked by comparison with the expression of 3 amplified multi-tissue samples (3 independent pools of 12 tissues = MT) and non-amplified individual tissues. X axis from the left: PDO, PMO, SCO, SAO, PDG, PMG, SCG, SAG, MT1, MT2, MT3, fetal ovary, pituitary gland, hypothalamus, muscle, skin, hurt, lung, intestine, stomach, liver, kidney, spleen, fetal ovary, theca. The left Y axis corresponds to the relative expression normalized by 2 reference genes (*Actin β* and *RPL19)* from qPCR data (red color). The right Y axis corresponds to the normalized counts from RNA-seq data (blue color).

Among these 15 349 expressed genes, we observed a significant decrease in oocyte-expressed genes during early follicular development, i.e., from 13 219 genes in primordial oocytes to 11 693 genes in small antrum oocytes (Figure 2A). Conjointly 9 499 genes were found to be expressed in all the samples (O + GCs + MT) and were considered as ubiquitously expressed genes. However, their relative expression levels varied depending on the type of cell, for example ribosomal genes (*RPL4*, *RPL14*), *VIM*, *SPARC,* and low expressed genes such as *BSCL2*.

In addition to this qualitative assessment, the transcriptome data set was analyzed using the R software package, DESeq [13]. Principal component analysis of the DESeq normalized data confirmed the relevance of the data (Figure 2B). The first axis explained 52% of expression variability and separated the two follicular compartments (O and GC). The second axis explained 15% of expression variability and separated the samples according to their follicular stages.

### Global differential gene expression

To identify differentially expressed genes, a generalized linear model was applied to the data after DEseq normalization (DESeq’s GLM method using the gene data set). This model allowed statistical evaluation of the factor effects (O, GC) and their interactions. The following criteria including FDR < 0.5% to 1% (Benjamini-Hochberg procedure) and a fold change >2 were chosen to determine significant differentially expressed genes. Using these criteria, 33.4% of genes (FDR < 0.5%: 5 130 genes) were significantly differentially expressed between the two compartments; 19.6% of genes were significantly differentially expressed during early development (FDR < 1%, 3 015 genes). We observed differences in expression profiles during early folliculogenesis between the two compartments for 4.3% of the genes (interaction effect FDR < 0.5%, 674 genes). For example, the expression of the *KCNN3* gene decreased in GCs during early development whereas its expression increased in oocytes. Differential expression is detailed in Table 1, which, interestingly, shows that genes were under expressed in oocytes compared to in GCs (2 833 genes).

**Table 1:** Summary of differential gene expression

		**Number of differential genes**		
		**Total**	**gene regulation**	
			**Up**	**Down**
Compartment effect: O/GCs		5129	2297	2832
Stage effect: SA/PD	total	3015	1357	1658
	oocyte	2173	1064	1109
	granulosa cells	1192	408	784
Interaction effect	total	674		
	oocyte	522	250	272
	granulosa cells	262	91	171

### Focus on differential expression between compartments (O/GCs)

We focused our analysis on the 5 130 significant differentially expressed genes (DEG) between compartments (Additional file 3). The aim was to provide an overview of specific gene expression, mechanisms and functions in these cell types, which may be involved in the dialog between the oocyte and GCs, and regulate follicle development.

First, we observed numerous genes on the DEG list already known to have cell-type specific expression. The expression of 39 genes known to be compartment specific was investigated and the expression of 36 of them was confirmed by the RNA-seq statistical analysis (Additional file 4).

Last, the expression of 33% of the genes (1 694 genes) varied during early follicular development. Supervised hierarchical clustering was performed to identify groups of genes with similar expression profiles. This led to a clear separation between the two compartments and identified clusters of genes according to the stage of follicular development, suggesting a difference in the dynamics of transcription between oocytes and GCs (Figure 2C).

### Compartment specific expression

Previous studies that were mainly devoted to rodent species identified oocyte-specific genes with important functions related to oocyte development and folliculogenesis. However, few data are available for GCs. To gain insight into the specific expression of each compartment, we performed a pair-wise comparison of the transcriptome of each compartment and both MT and the other compartment transcriptomes. In this way, we identified 55 highest differentially expressed genes in GCs (FDR < 0.5%, FC > 5) and 161 highest differentially expressed genes in oocytes (FDR < 0.5%, FC > 10. In so doing, we confirmed the specific expression of the genes already known as *WNT4*, *FSHR*, *PTEN* in GCs or *WEE2*, *DDX4*, *NRLP9*, *NLRP13*, *BMP15* in oocytes. We also identified new compartment-specific expression of genes such as *IFNE*, *DEFB112*, *BMP1*, *IGFBP6* and *TCF23* in GCs and *ST8SIA3*, *MUSK*, *TECTA*, *BTG4* and *ACCSL* in oocytes. Among these genes, we also identified oocyte-specific expression of 11 transcription regulators (*BSX*, *PATL2*, *DMRTC2*, *SIX6*, etc.) and the GC-restricted expression of four transcription regulators (*HDAC9*, *LZTS1*, *HOXC4*, *ASB9*). For a list of the highest differentially expressed genes, see Additional file 5.

### Validation of differential expression by quantitative real time RT-PCR

To validate the RNA-seq analysis, the expression profiles of a subset of genes of interest were monitored using qRT-PCR. These genes were selected as follows: (i) genes showing significant up or down expression in oocytes (11 genes: Table 2), (ii) genes involved in particular canonical pathways (2 genes: Table 2) or (iii) compartment-specifically expressed genes (17 genes: 8 oocyte-specific genes and 9 GC-specific genes; Table 3). Statistical analysis confirmed the differential expression between compartments observed in RNA-seq data for all the genes tested (Table 2) and the compartment-specific expression of 13 out of 17 genes (Table 3). The accuracy of our gene expression measurement was also confirmed by the similarity between RNA-seq and qRT-PCR fold change (Additional file 1: Figure S4). Figure 2D illustrates the new compartment-specific expressions checked by qRT-PCR in oocytes (*BTG4, MCF2L, ST8SIA3)* and in GCs *(IFNE, ITIH5, DEFB112).*

**Table 2:** qRT-PCR validation

**Canonical pathways**	**Gene**	**RNA-seq analysis**	**qPCR analysis**	
		**O/GCs Fold change**	**O/GCs Fold change**	**P value class**
IGF1	*IGF1*	0.284	0.52	*
	*INSL3*	0.121	0.482	*
	*IGF1R*	3.334	7.35	*
Gap junction	*GJA4*	20.84	4.247	*
	*GJA1*	0.117	0.278	***
Notch	*JAG1*	2.053	2.909	***
PI3K	*PIK3R3*	P	P	
	*KITLG*	0.266	0.218	**
	*KIT*	2.244	3.962	**
Paxilin	*PARVA*	P	3.5	*
Others	*GATA2*	7.028	10.906	*
	*AMHR2*	+∞	0.64	***
	*MAEL*	3.255	5.661	***

*: pval < 0.05; **: pval <0.01; ***: pval < 0.005.

**Table 3:** Validation of gene expression specificity

	**RNAseq data**		**qPCR data**	
**HUGO gene name**	**O/GCs FC**	**Pvalue**	**O/G FC**	**Pvalue**
*WEE2*	40.25	6.46E-15	56.15	*
*MUSK*	22.52	1.40E-10	108.64	*
*SPO11*	13.16	7.71E-06	16.45	***
*GAPT*	+ 999^1^	2.5E-05	60.91	*
*MCF2L*	41.16	3.82E-03	29.04	NS(0.068)
*ST8SIA3*	16.54	4.4E-03	74.5	*
*TNIP3*	41.79	1.1E-03	21.74	NS(0.072)
*GABRP*	28.89	1.79E-07	1369.57	*
*IFNE*	−999^2^	5.18E-05	0.002	*
*TCF23*	0.096	5.29E −08	0.051	***
*TLL2*	0.118	3.00E-11	0.148	*
*EFS*	0.086	5.39E-06	0.24	***
*WINT4*	0.095	4.63E −03	0.106	*
*ITIH5*	0.142	4.97E −03	0.101	***
*HTRIF*	0.007	2.21E-06	0	
*TMEM167A*	0.012	8.16E −04	0.025	
*DEFB112*	0.087	1.60E-12	0.005	*

*: pval < 0.05; ***: pval < 0.005.

^1^: expression only detected in oocytes.

^2^: expression only detected in GCs.

### Biological trends

#### Biological functions

**Figure 3:**
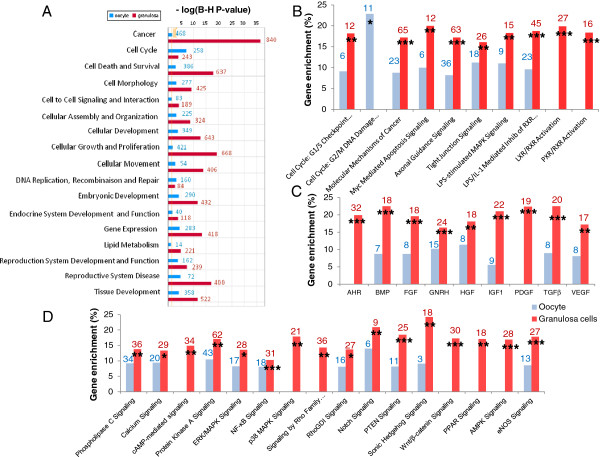
**Functional enrichment and canonical pathways within oocyte and GCs. A**: Functional enrichment. Genes differentially expressed between the 2 compartments were evaluated *in silico* using Ingenuity Pathway Analysis (IPA). The figure shows the most significant functional groups (p < 0.05, FDR < 5%). For each compartment, the gene list corresponds to up regulated genes compared to the other compartments. The bars represent the p-value at *logarithmic* scale. GC data are in red and oocyte data are in blue. Red and blue numbers correspond to the number of focus genes that contributed to the function concerned. **B**: Canonical pathways in oocytes and GCs. Significantly enriched canonical pathway categories (p-value < 0.05) identified in the 2 compartments. The Y axis corresponds to percentage gene enrichment in the pathway. Red and blue numbers correspond to the number of focus genes that contributed to the pathway concerned. *: FDR < 0.25 (likely to be valid 3 out of 4 times); **: FDR <0.10; ***: FDR < 0.05. **C**: Growth factor signaling pathways in oocytes and GCs. Significantly enriched growth factor signaling pathways (p-value < 0.05) identified in the 2 compartments. The Y axis corresponds to percentage gene enrichment in the pathway. Red and blue numbers correspond to the number of focus genes that contributed to the pathway concerned. *: FDR < 0.25; **: FDR <0.10; ***: FDR < 0.05. **D**: Signaling pathway in oocytes and GCs. Significantly enriched signaling pathways (p-value < 0.05) identified in the 2 compartments. The Y axis corresponds to percentage gene enrichment in the pathway. Red and blue numbers correspond to the number of focus genes that contributed to the pathway concerned. *: FDR < 0.25; **: FDR <0.10; ***: FDR < 0.05.

To investigate the biological functions associated with the two compartments, the DEG list was subjected to Ingenuity Pathway Analysis (IPA) and tested for function enrichment (Additional files 6 and 7). In the oocyte compartment (2 297 genes), we found a high statistical enrichment of genes involved in the cell cycle (with 14 genes contributing to the meiotic arrest), gene regulation, cell morphology and organization, and the reproductive system. The cell cycle function (mainly cell cycle progression) was also enriched in GCs. Last, we observed different involvement of oocytes and GCs in the reproductive system. On one hand, oocytes over-expressed 66 genes that have been shown to play important roles in fertility. The genes over-expressed in GCs (2 833 genes) were found to be mainly related to cellular proliferation and atresia (“cellular growth and proliferation”, “cell death” IPA functions), cell morphology (148 genes involved in the “formation of protrusions”) and movement, lipid metabolism and the reproductive system such as *BMP15*, *GDF9*, *BOLL*, *FIGLA*, *MOS*, *PIWIL1*, *INSL6*. On the other hand, GCs over-expressed 107 genes involved in ovarian tumors of which eight were related to GC cancer (*AMH*, *FOXL2*, *GATA4*, *GNAS*, *INHA*, *KRAS*, *NR5A1*, *ZFPM2*). Figure 3A is a graphic representation of the DEG biological functions in which the 17 main categories (FDR <0.05) are shown.

#### Canonical pathways

Based on the results of the IPA canonical pathway analysis, we identified 36 significant pathways that encompassed metabolism, regulatory and cell signaling. These pathways are shown in Figures 3B-D. This analysis underlined two different cell cycle checkpoint regulations according to the compartment: enrichment of genes involved in the G1/S checkpoint for GCs and in the G2/M DNA damage checkpoint for oocytes (Figure 3B). We observed significant cellular proliferation and apoptosis mechanisms in GCs, and gene enrichment in nuclear signaling receptors such as PPARG, LRX/RXR and PRX/RXR involved in lipid metabolism. We also identified GC-specific enrichment pathways such as cAMP-mediated, p38 MAPK, wnt/β-catenin and PDGF signaling pathways. Figures 3C-D provide further evidence for the over-expression of growth hormone-encoding genes in GCs compared to oocytes during early ovarian folliculogenesis, and for the involvement of PTEN, calcium, eNos and SHH signaling pathways in GCs. Last, we observed enrichment of genes involved in AMPK signaling related to GC energy regulation.

#### Cell-cell interactions

**Figure 4:**
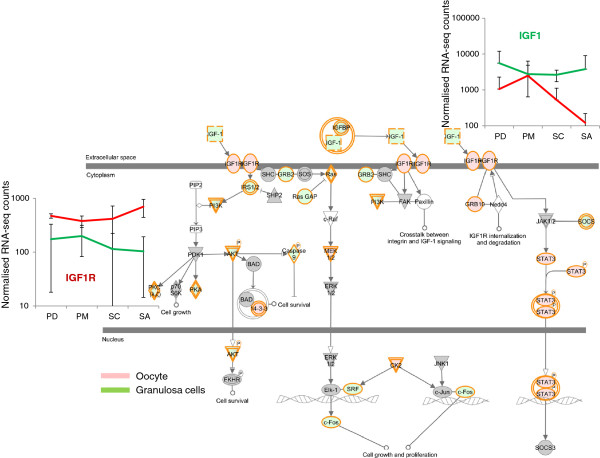
**IGF signaling pathway.** Gene expression involved in the IGF1 signaling pathway. Genes in red are over expressed in oocytes. Genes in green are over expressed in GCs. Relative expression of IGF1 and IGF1R from RNAseq data in the graph. The Y axis corresponds to RNA-seq normalized read counts.

In mammals, bidirectional communications between oocyte-GC and GC-GC are required for meiotic maturation, acquisition of oocyte competence, and follicle development. Analysis of the canonical pathways identified molecular events involved in these communications including IGF1, VEGF, FGF, Notch and tight junction signaling pathways. Figure 4 illustrates oocyte-GC cross-talk with the over expression of *IGF1R* gene in oocytes (FC = 3.33) and the over expression of *IGF1* gene in GCs (FC = 3.57).

**Figure 5:**
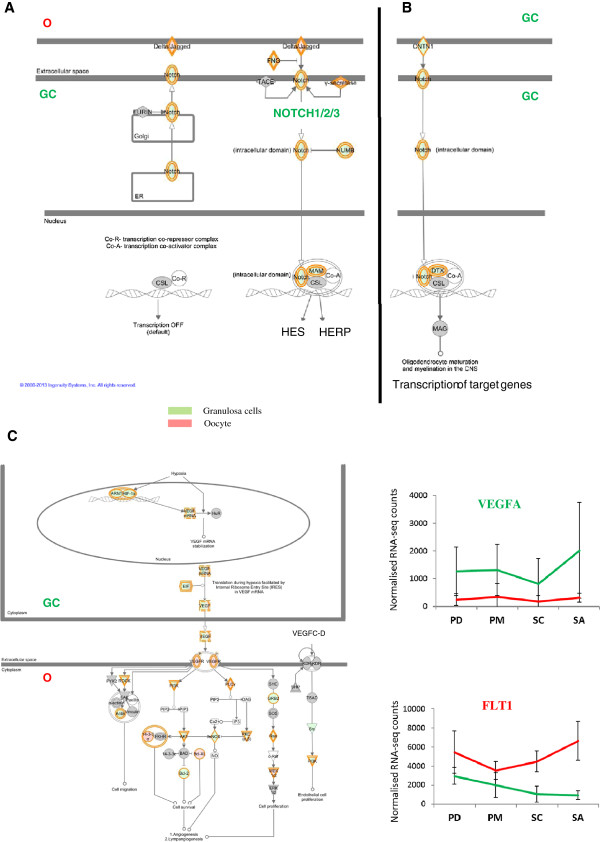
**VEGF and NOTCH signaling pathways.** Gene expression involved in the VEGF and NOTCH signaling pathways. Genes in red are over expressed in oocytes. Genes in green are over expressed in GCs. Relative expression of VEGF and FLT1 from RNAseq data. The Y axis corresponds to RNA-seq normalized read counts. **A**: Oocyte-GC communications in the NOTCH signaling pathway. **B**: GC-GC communications in the NOTCH signaling pathway. **C**: Oocyte-GC communications in the VEGF signaling pathway.

Regulation of the IGF1 system was then achieved through the over expression of *IGFBP2*, *IGFBP4*, *IGFBP6*, *IGFBP7* genes in GCs. In the same way, *VEGFA* was highly expressed in GCs (FC = 4.87) while its receptor *FLT1* was over expressed in oocytes (FC = 2.86) (Figure 5A). The involvement of *FGF2* in oocyte-GC dialog was also evidenced by the over expression of *FGF2* in oocytes and of *FGFR1* and *FGFR2* in GCs. We also revealed for the first time over expression of FGF family members such as *FGF16* (FC = 7.14), *FGF20* (FC = 4.3) in oocytes and *FGF1* (FC = 8.3), *FGF11* (FC = 4.3), *FGF12* (FC = 6) in GCs. Notch signaling was assumed to be involved in two kinds of interactions. Firstly, the abundance of *NOTCH1* transcripts in GCs and of its ligand *JAG1* in oocyte confirmed compartmental interactions (Figure 5B). Secondly, this pathway could be involved in GC interactions via its functional ligand CNTN1 (Figure 5C).

Finally, we detected the differential expression of genes involved in cellular connections and adhesions. *GJA1* (*cx43*), which is over expressed in GCs, and *GJA4* (*cx37*), which is over expressed in oocytes (Table 2), are known to participate to gap junctions and connect oocytes with GCs. We also observed over expression of genes involved in the adherent junctions (catenin, cadherins and afadin) and in the tight junctions (*TJP1*, claudins, cingulin) (Additional file 1: Figure S5).

#### Transcription regulators

Oocyte and GC transcription factors (*FIGLA*, *NOBOX*, *FOXL2*, etc.) play an important role in folliculogenesis [14]. They control both oocyte development and GC function. To identify new transcription regulators, we performed IPA upstream regulator analysis on the DEG data set. For each compartment, we predicted the transcriptional regulators that were over expressed and involved in the regulation of this DEG: 47 GC regulators and 9 oocyte regulators (pval < 0.05: Additional file 8). Among them, the oocyte transcription regulator NOBOX and 12 GC transcription regulators (GATA4, FOS, TP53, etc.) were predicted to have their protein activated.

## Discussion

To our knowledge, this is the first comprehensive exploration of transcriptome from oocyte and granulosa cells separately and at several key stages corresponding to *in vivo* major transitions during early folliculogenesis in sheep. The study resulted in reliable transcriptional profiling in time and space. We explored changes in gene expression between the two follicular compartments and identified specific transcriptional programs in either oocyte or granulosa cells. In addition, we identified pathways involved in bidirectional communication.

Thanks to mouse genetic models, a significant number of targeted molecular data (gene expression and proteins) has been accumulated during the past years. Recently, these data were complemented by mouse and human oocyte transcriptome data that give us more information about the molecular mechanisms involved in early folliculogenesis. Our study expanded transcriptome data to GC and enabled us to determine the precise expression of genes involved in these mechanisms in a mono ovulating species.

### Oocyte and GC Transcriptional profiling

This study documented the expression of 15 349 genes (which represents the majority of ovine genes) in ovarian follicles during early follicular development. Thanks to the sensitivity of RNAseq, we estimated a larger number of genes expressed in oocytes (14 172 genes expressed in ¾ of replicates) than other microarray studies (Additional file 2; discussion section). In our study, 14 087 genes were also estimated to be expressed in GCs. These high numbers of expressed genes were close to the higher number of genes expressed in testis (15 000) compared to the other human and mouse tissues (11 000 to 13 000 genes from RNA-seq data) [15].

The high number of expressed genes in both oocytes and GC evidenced strong transcriptional activity. Interestingly, this analysis revealed a slight decrease in the number of oocyte expressed genes during follicular development from SC follicles that coincides with the gain in methylation from PM to SC follicle transition (oocyte diameter of at least ~50 mm) [16]. The detection of a large number of ubiquitous genes (Figure 2A) was previously reported by Ramköld et al. [15] suggesting that functional specialization is not associated with a large population of genes but results in more subtle and complex control.

RNA-seq has made it possible to identify novel transcripts. In this study, we uncovered 221 716 genomic fragments that now need to be annotated. Some of them present a high expression level and compartment-specific expression (FC > 100) suggesting they play an important role in early folliculogenesis. Further exploration using bioinformatics and gene expression validation will be necessary to refine these results.

### Differential expression between compartments reflects the fate of the cell types

The development of the follicle and the maturation of oocyte require orchestrated communication between oocytes and GCs at all stages of early folliculogenesis. They also require the expression of compartment-specific genes. To investigate these molecular processes, we focused our analysis on differences in gene expression between oocytes and GCs and identified a large number (5 129) of differentially expressed genes. We identified functional differences between oocytes and GCs and described the characteristics of each compartment that lead to two specific cell fates. As described in our previous study [12], the top functions of differentially expressed genes illustrated the three main cellular events known to be involved in early follicular development: the change in the shape of the GC cell from flattened to cuboidal (IPA categories “cell movement”; “shape change of tumor cell lines”: 30 genes including *FLNB*, *EDNRA*), the marked increase in the number of GCs (x 40 in sheep species up to the secondary stage) and oocyte enlargement [17] (x 300 between the primordial and antral stages: IPA category “morphology of germ cells”: 31 genes including *ASZ1*, *TDRD5*). DEG comparison of genes identified in five previous studies validated this list (Additional file 2; discussion section). In addition to the information provided by our previous study [12], IPA RNA-seq analysis increased available information thanks to the identification of additional DEG.

### Meiotic regulation

In most mammals, meiosis is initiated during fetal life and stops prenatally in the diplotene stage of prophase I at the G2/M transition until development into a Graafian follicle (before ovulation).

This study highlights oocyte over expression of genes involved in the regulation of meiotic progression, acquisition of meiotic maturation, and maintenance of meiotic arrest.

For instance, SPO11 is involved in initiating meiotic recombination by catalyzing DNA double strand breaks [18]*. BTRC* loss of function in mice results in slow progression through meiosis and abnormal divisions in spermatocytes [19]. We also observed a significant increase in sheep *OVOL1* mRNA (FDR = 0.043) and *ASZ1* mRNA (FDR = 0.017) during early folliculogenesis. Ovol1 is known to be involved in regulating the pachytene progression of male germ cells [20] and shown to be expressed during sheep early folliculogenesis [21]. In mice, the ASZ1 protein is a germ cell-specific protein located in the cytoplasm of oocytes at all stages of development that may act as an important signaling protein and/or transcriptional regulator during germ cell maturation [22].

The function of these genes is poorly understood in mammalian ovary development but their expression in early follicular development suggests they play a role in the regulation of female meiosis.

Finally, we confirmed the expression of genes already known to be involved in the regulation of the oocyte and/or spermatocyte meiotic arrest (*AURKA*-C, *PLK1*, *PDE3A, BTRC, CDC25B*-*C, WEE2* etc*.*).

### The establishment of GC steroid synthesis

This study clearly shows that GCs over-expressed genes involved in steroid synthesis (such as *CYP11A1*, *HSD3B* and *HSD17)* and in steroid regulation (such as nuclear receptor genes (NRs)). NRs include orphan receptors (*NR5A1*, *NROB2*), steroid hormone receptors (*ESR1*, *AR*, *LXR* (*NR1H2*), *FXR (NR1H4)),* and retinoid acid receptors (*RXRA),* all of which are over expressed in GCs during preantral follicle stages*.*

*NR5A1* (*SF-1*), which has already been described in sheep GCs [23], is a master gene that regulates the expression of numerous genes including reproductive and steroidogenic enzyme-encoding genes [24]. GC specific SF-1 KO mice are infertile whose ovaries contain a reduced number of follicles and reduced expression of *AMH*, which partially correlates with the reserve of ovarian follicles [25].

E2 and androgens hormones exert their influence on early folliculogenesis via their respective receptors, ESR and AR. Like in mice, E2 probably plays a role in controlling the primordial follicle pool [26] and androgen signaling is crucial for normal folliculogenesis [27]. We observed strong expression of *AR* in GCs, like in humans [28]. Finally, for the first time, we identified over expression of *LXRB, FXRA* and *RXRA* in GC preantral stages. RXR is a retinoid X receptor that binds as heterodimers (LXR/RXR, FXR/RXR…) and becomes transcriptionally active only in the presence of a ligand (RXR-selective ligand or ligands of the other dimer). LXRs are activated by oxysterols (generated by intermediates of steroid hormone and cholesterol synthesis (CYP21A1, STAR, P450scc products)) and are key regulators of ovarian steroidogenesis at antral stages [29]. FXRs are activated by bile acid, sterol and different lipids and are possibly important regulators of androgen homoeostasis in male gonads [30].

### Complex regulation of follicular atresia

Atresia is an important process in folliculogenesis and concerns the majority of the follicles. Apoptosis is found in the oocytes of primordial follicles and progressively extends to GCs of growing follicles.

We characterized the expression of different genes of the BCL2 family either over expressed in oocytes (*BCL2L1, BCL2L10, BCL2L11* (*BIM*), *BCL2L14 (BCLG)*) or in GCs (*BCL2 BCL2L2, BOK*) and that mainly prevent oocyte loss. For example, the *BCL2L1* gene was shown to play a crucial role in the survival of germ cells [31]. BCL2L10 may antagonize BAX activities and may also play other roles related to cell cycle control and oocyte maturation (for a review see [32]). In sheep, the high expression of *BCL2L10* in oocytes and its up-regulation during preantral stages reinforces its already important role in oocyte development. By decreasing GC atresia, the *BCL2* gene family expressed in GCs, such as *BCL2*, was also believed to increase the number of germ cells and enhance their development. Lastly, *BOK* mRNA, was found to be restricted to reproductive tissue and was detected in GCs from PM to antral follicles in rat species [33].

### GC signaling pathways

The molecular pathways were mainly linked to GC proliferation, steroid production, and morphological change.

Sheep GCs over expressed members of the Sonic hedgehog (Shh) signaling pathway (*SMO, PTCH1* and *GLI1-2-3)*, which is consistent with a potential role for the HH pathway in increased GC proliferation during preantral stages [34]. Last, genes of the WNT family and their receptors (*WNT4-3A-5A-6*, *FDZ1*, *LRP1-11*) and Rho-GTPase activating proteins (*ARHGAP9-20-21-31-36-44*) were shown to be over expressed in GCs. In the ovary, the β-catenin dependant canonical pathway of WNT4 leads to ovarian determining pathways. WNT proteins can also signal via Rho-GTPases, using non-canonical pathways that involve changes in polarized cell shape and cell migrations [35]. This pathway may promote changes in cell shape that affect GCs during early folliculogenesis.

### Cell-cell communications

Follicular growth depends on close interactions between oocytes and GCs but also between GCs themselves. Two kinds of cell interactions are described in this study demonstrating the existence of complex dialogs between compartments: a molecular dialog (signaling pathways) and physical communications (gap junctions and trans-zonal projections).

#### Signaling pathways: GC regulation of oocyte development

GCs are known to play a role in supporting the growth of oocytes, regulating the progression of meiosis, and modulating global oocyte transcription activity. Nevertheless, so far, few factors have been identified that support these assumptions. To date, the most widely studied ligand-receptor systems (that were also identified in the present study) are the KIT/KITL and BMP pathways. Below we describe the involvement of other signaling pathways.

The Notch signaling pathway plays many roles in cell communication by influencing cell proliferation, differentiation, and apoptosis [36]. In sheep, we found over expression of *NOTCH1-2-3* in GCs and over expression of the NOTCH ligands *JAG1* and *DLL3* in oocytes that perfectly illustrate oocyte-GC crosstalk (Figure 5A) and that may influence oocyte apoptosis, like in postnatal mice [37]. In addition, we suggest that NOTCH is involved in GC proliferation [38] through NOTCH1/CNTN1 binding (Figure 5B).

Vascular endothelial growth factor (VEGF) was already known to promote early folliculogenesis [39]. In this study, we identified over expression of members of VEGF pathway including *VEGFA* and *NRP1* (a VEGF receptor) in GCs, and over expression of *FLT1* (another VEGF receptor) in oocytes. These gene expressions suggest a role for the VEGF pathway in GC-oocyte and GC-GC crosstalks (Figure 5C). As already described, the VEGF pathway may protect GC against atresia [40].

The crucial role of the IGF system is largely described during terminal follicular growth [41] but is poorly documented in preantral stages. Ovarian IGF1 production was shown to vary with the species. IGF1 is mainly expressed by GCs in rat and pig species [42] but its protein was also detected in the oocyte of rat preantral follicles [43]. Moreover, the *IGF1* transcript was not detected in adult sheep [44] or cattle ovaries [42] but its receptor IGF1R was expressed from primary follicles and its expression increased with follicular growth [44,45]. Our study provides a precise description of members of the IGF system in sheep species at preantral stages (*IGF1-2*, *IGFBPs*, *IGF1R*). The IGF1 pathway (Figure 4) suggests a role for IGF1 in oocytes and GCs in agreement with studies on mice. Indeed, although insulin/IGF signaling is not essential in oocyte conditional knockout mice [46], IGF-1 significantly increases oocyte maturation [47]. In GCs, the expression of *IGFR1* and the over expression of the downstream signaling molecules *IRS1* and *AKT2* are also in agreement with the involvement of IGF1 in the regulation of GC survival [48]. Additional immunohistochemistry to detect IGF1 and IGF1R proteins and *in vitro* early follicle cultures could be used to evaluate the effect of IGF1 on sheep preantral growth. *In vitro* early follicle cultures are described in Mochida et al [49].

#### Signaling pathways: Oocyte regulation of GC function

Numerous studies have shown that oocytes are potent stimulators of GC proliferation. A compilation of oocyte-secreted factors that regulate GC function was published in 2008 [50]. Among these factors, we describe the over expression of *FGF2* in sheep oocytes and *FGFR1-2-L1* receptors in GCs that may influence primordial follicle development in accordance with *in vitro* goat and rat studies [51,52]. We also report the over expression of *FGF16-20* in oocytes for the first time in mammals. A recent study on Nile tilapia species suggests that FGF16-20 are involved in early oocyte development in the female [53]. In other mammal tissues, FGF16 and FGF20 promote cell proliferation and differentiation.

#### Physical interactions

Transzonal projections (TZP) are GC specialized extensions corresponding to long cell projections and microvilli that are in close contact with the surface of the oolemma or invaginate the cortical area of the oocyte. These projections establish communicative (gap junctions) and adhesive junctions (tight junctions) both with the oocyte and neighboring transzonal projections. In this study, we observed an enrichment of genes involved in the formation of cellular protrusions in both GCs and oocytes (Additional files 5 and 6). We confirmed over expression of two gap junction genes that are indispensable for the regulation of folliculogenesis and oogenesis: *GJA1* (*Cx43*) in GCs and *GJA4 (Cx37)* in oocytes. Moreover in our study, *ZP2-4* were seen to significantly increase during preantral stages as shown in human fetal ovaries before the formation of *zona pellicida*[54].

### Regulators

In the present experiment, we identified a set of upstream transcriptional regulators able to regulate follicle gene expression and ovarian development.

In sheep oocytes, *NOBOX*, which is known to promote follicular growth in mice, was detected in this study at medium expression level in preantral oocytes and was predicted to increase gene expression of *BMP15*, *DNMT1*, *GDF9*, *H1FOO*, *MOS*, *ZAR1* genes [55]. Analysis of IPA upstream regulators also predicted activation of AHR in oocytes (Additional file 9). AHR protein may act on oocyte gene expression through the receptor nuclear translocator *ARNTL2* over expressed in oocytes. These findings are consistent with AHR regulation of follicular growth by affecting germ cell death during female gametogenesis [56].

In GCs, transcriptional regulators referred to sterol regulation (*SREBF1*, *SREBF2*) hormone response (*EGR1*, *CREB*, *SKIL*, *ETS1*), cellular growth (*TP53*, *XBP1*, *MYC*, NFIA, *HIF1A*), cellular differentiation (*MITF*), and increased transcription (*MYC*, *NFIA*). Here we describe for the first time, over expression of *NFIA* and *HIF1A* in GCs of preantral follicles. NFIA is a transcription factor involved in the control of cell growth in both humans and model systems [57]. HIF1A is an ARNT interacting protein that activates the transcription of target genes involved in energy metabolism, angiogenesis, and apoptosis [58]. We were able to predict the regulation of 59 genes by *HIF1A* and we observed an increased in its expression during early folliculogenesis, assuming it plays a role in preantral development.

### Genes preferentially expressed according to cell type

Oocyte-specific genes and their role in folliculogenesis have been extensively studied in murine knockout models [59] but there is a relative paucity of information concerning GC-specific genes. In this study, we completed existing data by highlighting the expression of 216 preferentially expressed genes (161 O + 55 GCs), including genes whose function in ovarian folliculogenesis is unknown. Nevertheless, GCs exhibited fewer preferentially expressed genes than oocytes.

In GCs, the *DEFB112* gene is described for the first time as preferentially expressed. This gene is poorly documented but is reported to have a regionalized expression in the epididymis in rat and in human and to be related to sperm maturation [60]. As already described in sheep, the preferential expression of *BMP1*, which promotes BMP signaling via chordinase activities [61], was confirmed. Finally, the molecular chaperone *TCP1*, which assists the folding of proteins upon ATP hydrolysis (such as actin and tubulin), was specifically detected at a slight but constant level of expression during the preantral stage. TCP1 appears to play a key role in the cytodifferentiation of spermatids that relies on the high plasticity of microtubules [62]. Our result may be associated to the remodeling of the microtubule cytoskeleton during the change in cell shape.

In oocytes, we identified the preferential expression of genes such as *BTG4*. Although much about the function of BTG4 remains unknown, it is known to be involved in cell cycle arrest and to be endowed with antiproliferative properties. In mice, its expression is restricted to the olfactory epithelium, testis, and oocyte [63]. The role of *BTG4* during preantral stages in sheep requires further investigation. Another gene of interest is *PATL2*, a RNA-binding protein (called P100) that was originally characterized as being oocyte specific in *Xenopus laevis,* where it acts as a translational repressor in a CPEB-containing complex [64]. In Xenopus *laevis*, *PATL2* is maternally expressed in immature oocytes, but disappears upon meiotic maturation. Some authors suggest that PATL2 plays a role in regulating the translation of specific maternal mRNAs required for the progression of *Xenopus laevis* oocyte maturation [65]. The expression of *PATL2* has not yet been documented in mammals; but here we describe for the first time increased specific expression of this gene in oocytes during preantral stages in sheep. In comparison with the role of *PATL2* in *Xenopus laevis*, our finding suggests it plays an important role in the regulation of the translation in preantral oocytes in sheep. Finally, we identified over expression of *MUSK* in oocytes at preantral stages. MUSK is a receptor tyrosine kinase (RTK) which is induced during skeletal muscle differentiation, and is dramatically down-regulated in mature muscle [66].

This study highlighted involvement of new gene expression in early folliculogenesis. Further investigations are underway to estimate the implication of these genes in mammalian folliculogenesis.

## Conclusions

This work generated a significant amount of expression data that accurately document early folliculogenesis in sheep. The abundance of expressed genes and the differential expression profiles found in oocytes confirm the hypothesis that oocytes are dynamic cells. The decrease in the number of expressed genes following the development of oocytes is in accordance with a general decrease in transcription.

This study identified specific functions and molecular mechanisms involved in each cell type during early folliculogenesis and in the dialog between the different cell types. We confirm and incorporate a large number of individual studies in different species. Last, we highlight new gene expressions that support and extend current knowledge on early folliculogenesis.

This study is the first thorough, integrated overview of the molecular mechanisms involved in early folliculogenesis in a mono-ovulating species. It provides a valuable data base for future functional studies in mono-ovulating species including humans.

## Methods

### Laser capture microdissection

Ovaries were sampled during the preliminary experiment [12] which was conducted in compliance with institutional guidelines for research studies (animal experimentation authorization no 31–297, prefecture de la Haute Garonne). As described in that study, the ovaries were removed from lambs at birth and embedded in O.C.T. embedding matrix, frozen in liquid nitrogen and stored at −80°C until use. LCM capture was performed using the Arcturus XT apparatus (Arcturus, Applied Biosystems®) from eight-micrometer frozen sections that were stained individually using Cresyl Violet.

Each follicle stage was selected as a function of follicular diameter under the microscope taking the observation of series of sections into account and according to the classification of Lundy *et al.*[17]. Briefly, the following criteria were applied for the selection of the follicle stage : i) primordial follicles (PD <35 μM) were defined as an oocyte surrounded by 2–3 flattened GCs and no cuboidal GCs, ii) primary follicles (PM: 35-50 μM) were defined as a monolayer of cuboidal GCs whatever the size of the oocyte, iii) secondary follicles (SC: 60-120 μM) were defined as two layers of GCs and iv) small antral follicles (SA: 250-500 μM) had a diameter of less than 500 μm. The follicular compartments (i.e*.* granulosa cells (GCs)) and oocytes (O) for each follicular stage were collected in separate CapSure™ HS LCM caps (Arcturus, Ref LCM 0214), treated with 10–15 μl of extraction buffer from Picopure RNA Isolation kit (Arcturus, ref: KIT0202) and stored at −80°C until use.

### RNA extraction

As very few cells were captured per cap, each RNA LCM-derived sample comprised pooled caps and was extracted using the PicoPure RNA Isolation Kit according to the manufacturer’s instructions, including on-column DNase treatment (Qiagen, ref 79254, Courtaboeuf, France) [12]. Finally, RNA of three/four independent replicates was extracted per biological condition (O and GC compartment from PD, PM, SC, SA stages).

The 13 tissue samples (fetal ovary, pituitary gland, hypothalamus, muscle, skin, heart, lung, intestine, stomach, kidney, spleen, liver, and theca) were sampled in triplicate at the local slaughterhouse (3 individual animals), frozen in liquid nitrogen and stored at −80°C until RNA extraction. Total RNA was isolated using the Nucleospin RNA II kit (Macherey-Nagel GmbH & Co, ref 740 955 50, HOERDT, France). The 12 total RNA tissue samples (except fetal ovary) were pooled in three independent groups.

### T7 linear amplification

A total of 31 RNA LCM-derived (3PDO, 4PMO, 4SCO, 4SAO, 4PDG, 4PMG, 4SCG and 4SAG) and three multi-tissue RNA samples were subjected to two rounds of T7 linear amplification using the RiboAmp®HS PLUS kit (Arcturus, ref KIT0525), as previously described [12]. After in vitro transcription, the optical density of antisense RNA (aRNA) was measured at 260 and 280 nm. The integrity of the aRNA samples was checked by the Agilent Bioanalyzer 2100 with a RNA 6000 Nano Lab Chip. To complete the check, we added four *Bacillus subtilis* control transcripts with decreasing quantities and transcript length (6.54 kb; 3.36 kb; 2.4 kb and 1.6 kb) (provided by Affymetrix, GeneChip Eukaryotic Poly-A RNA Control Kit, ref 900433) to each LCM-derived RNA and multi-tissue RNA sample before amplification (1 μl of 5*10-7 dilution). These control transcripts represented internal controls of gene expression for the experimental process.

### Library preparation and massive parallel sequencing

All RNA-seq libraries were constructed from 200 ng of aRNA using Illumina TruSeq RNA Sample Prep (Illumina, San Diego, CA USA) according to the manufacturer’s instructions (Additional file 1: Figure S1). Each biological replicate was identified by a different index. Libraries were quantified using the QPCR NGS Library Quant Kit (Agilent). Three libraries were pooled and loaded randomly per lane at a concentration of 8 pM. The PDG4 library was loaded twice as a technical replicate (PDG4, PDG4b). The 11 flow cell lanes were split into five runs (four runs for LCM derived RNA samples and one run for RNA multi-tissue samples). Sequencing was performed on an Illumina HiSeq2000 using the Illumina TruSeq SBS kit v2 (209 cycles including the index) to obtain paired-end reads (2x100 bp). The quality of sequencing results was summarized and plotted using the NG6 storage platform (http://ng6.toulouse.inra.fr) [67].

### Mapping strategy

The sheep genome sequence recently became available in public databases but the 3′ UTR non-coding sequences of ovine genes were still poorly documented. Due to the specificity of our data (enrichment of 3′UTR non-coding sequences [68]), we evaluated two different assembly strategies to maximize transcript identification (Additional file 1: Figure S6A).

#### Genome strategy

For each sample, the reads were mapped with bwa aln to the sheep genome sequence [69] (CSIRO Oarv2.0 released March 2011: http://www.livestockgenomics.csiro.au/sheep/). Bam files were filtered (keeping only reads in a proper pair with mapq > 30) and converted into BED files. All BED files were individually merged (overlapped genomic fragments or which shared reads from same pair) and then grouped before being merged to produce a collection of genomic fragments. The number of reads per genomic fragment was computed and only genomic fragments with more than 20 reads were kept to perform annotations.

#### *De novo* transcriptome strategy

Reads were assembled per condition using SOAPdenovo-Trans-31kmer. After each assembly, GapCloser was used to resolve N-spacers introduced in the scaffolding process. All contig files were thereafter concatenated and a meta-assembly was performed using TGICL. All contigs and singlets highlighted were used to create a reference to map back all input reads. Bam files were filtered to keep only reads in a proper pair with no more than four mismatches. A counting file was generated for each contig and each condition using all count files.

### Annotation strategy

In comparison to sheep, more gene annotations are available for cattle. In agreement with Jager [70], our preliminary analysis showed that the use of bovine transcripts for the annotation substantially increased the number of genes identified. Consequently, genomic fragments and *de novo* contigs were aligned with the bovine genome sequence [71,72] (Ensembl *Bos taurus* UMD3.1) using Blat. Blat results were filtered to keep only hits with genomic fragment or contig coverage > = 80%, identity > = 90% and best hit score 3 fold greater than other hits. Blat targets (i.e. cattle slices) were crossed with the Ensembl *Bos taurus* GTF file (Bos_taurus.UMD3.1.65.gtf). Iteratively, for *Bos taurus* slices not linked to a described gene, we searched for overlaps with downstream gene regions extended by 500 bp, 1 kb and 3 kb (Additional file 1: Figure S6B).

All the non-annotated genomic fragments and contigs (not mapped to the bovine transcript) were discarded from the data set. Finally, the annotated contigs (from the *de novo* strategy) were mapped to the sheep genome and contigs whose sequence was unknown in the public sheep genome database were added to the final data set.

### Dataset filtration

As a gene was represented by a mean of eight genomic fragments (Additional file 1: Figure S8) and because most of the RNA-seq reads are located at the end of the genes (amplification bias), the genomic dataset was filtered to conserve only the best genomic fragment per gene (the genomic fragment with the highest count and the most 3′ UTR localization) with more than 20–30 reads per condition (20 reads for PDO in the three replicates and 30 reads for the other conditions in the four replicates). This counting method was the most informative for this experiment and did not require gene length normalization. This genomic data set was completed with the annotated contigs from the *de novo* strategy missing in the genome strategy data set.

### Experimental validation

Increasing amounts of four *Bacillus subtilis* control transcripts were added to all LCM-derived RNA samples before amplification to control the amplification and RNA-seq processes. For each sample, the reads were mapped with bwa aln on the 4 *B. subtilis* sequences. The RPKM (reads per kilobase of exon per million) was computed for each control transcript. To make the samples comparable for experimental validation, the amount of each *B. subtilis* control transcript was calculated for each sample in proportion to the highest one (DAP).

### Statistical analysis

The significance of differential gene expression was determined using DESeq package for multifactor design [13] in R software (R 2.14.0; DESeq release 1.6.1) (Additional file 1: Figure S6C). Our experimental design included 2 factors: stage (with 4 levels: PD, PM, SC and SA) and compartment (2 levels: O, GCs) with interactions. After DESeq normalization, the best dispersion estimation of our data set was obtained using the following arguments: method ‘pooled’, sharingMode ‘fit-only’, fitType ‘local’. The GLM method was then used to estimate the coefficient and deviance for each gene. For inference, we specified three models: the full model regressing gene expression on both compartments and follicular stages and taking interaction effects into account (count ~ stage*compartment), the additive model (count ~ stage + compartment) and the reduced model (count ~ 1). We tested the effects of the compartments, follicle stages, and their interactions on gene expression using nbinomGLMTest. Differential gene expression between compartments and differential expression profiles between compartments (interaction effect) were selected with FDR < 0.5% (Benjamini-Hochberg procedure) and a fold change >2. To establish the differential expression profile during early follicular development, we selected genes whose intra-compartment variance was differentially expressed in at least one of the stages (nbinomGLMTest). Thereafter, for each compartment, the expression value of each gene at each follicle stage was compared using pairwise comparison (nbinomTest). Differential gene expression during early follicular development was selected with a FDR <5% for global ‘intra-compartment’ effect (nbinomGLMTest) and pval <1% for pairwise comparison (nbinomTest) with a fold change >2.

The most expressed genes in a specific compartment (O, GCs) were identified using pairwise comparisons with FDR <0.5% and a fold change >10 for the oocyte compartment (O/GCs + MT) and >5 for GC compartment (GCs/O + MT).

### Biological trends

Ingenuity® Pathway Analysis software (IPA; http://www.ingenuity.com) was used to examine functional and molecular pathway enrichment for differentially expressed genes based on Fisher’s exact test. This software combines functional annotations of differentially expressed genes (focus genes) and the corresponding bibliographic data to generate significant signaling pathways and regulation networks. The biological analysis focused on the list of differentially expressed genes. The Ingenuity Pathways Knowledge Base was used as the reference set.

Functional category analysis identified the functions in the IPA library that were most significant to the input data set. The significance of the association between the data set and the functional category was determined by a P value calculated using Fischer’s exact test corrected for multiple testing (p-value < 0.05; FDR < 0.05 (Benjamini-Hochberg test)).

Canonical pathway analysis identified the pathways in the IPA library of canonical pathways that were most significant to the input data set. The significance of the association between the data set and the canonical pathway was determined based on two parameters: (1) the ratio of the number of genes from the data set that mapped to the pathway divided by the total number of genes that mapped to the canonical pathway and (2) a P value calculated using Fischer’s exact test corrected for multiple testing to determine the probability that the association between the genes in the data set and the canonical pathway is due to chance alone (p-value < 0.05; FDR < 0.05-0.25 (FDR threshold <0.25 is proposed as appropriate for enrichment gene sets in BROAD GSEA analyses (http://www.broadinstitute.org/gsea/doc/GSEAUserGuideFrame.html). In these conditions, the result is likely to be valid 3 out of 4 times).

Downstream effects analysis was used to identify the expected effect of the observed change in gene expression on the functions (expected to increase or decrease). This analysis examined genes in our data set that are known to affect functions, compared the gene direction of change to expectations derived from the literature, and provided a prediction for each function based on the direction of change (in the list of differential gene expression). IPA uses the regulation z-score algorithm to make predictions.

In the same way, IPA upstream regulator analysis was used to understand the cause of the observed change in gene expression. This analysis identifies the cascade of upstream transcriptional regulators (any molecules that can affect the expression of other molecules) that can explain the observed change in gene expression in the dataset. The overlap pvalue measures statistically significant differences between genes in the data set and the genes that are regulated by a transcriptional regulator. The activator z-score infers the activation state to the transcriptional regulator (activator z-score < −2 and >2). Without identified bias, this z-score can always be used as an independent test to call downstream regulators. A bias means that the activation z-score and pvalue must be used to call downstream regulators. The expression and/or differential expression of the transcriptional regulator in the data set provides more evidence for the biological mechanism.

Combining oocyte and GC upstream effect/ regulator analyses provided clues to the putative regulation involved in the oocyte-GC dialog.

### Quantitative real time RT-PCR analysis for gene expression

Gene primers were designed from RNA-seq sequences (Additional file 10). The intron–exon organization of ovine genes was deduced by comparison with the Human genome using the NCBI database. Gene primers were designed preferentially in the 3′ UTR (last 1000 base pairs) or the last exon using LightCycler Probe Design2 software (Roche Diagnostics). The primer pairs were confirmed by Primer3 (http://frodo.wi.mit.edu/primer3/). Sequences are available in Additional file 8.

The LCM-derived aRNA samples were reverse transcribed as previously described [12]. The resulting LCM-derived cDNA samples were completed to 50 μl and diluted 1/20 before PCR. The assay for each gene consisted of four replicates per condition (except for PDO = 3) and negative controls.

Total tissue RNA samples (2 μg) were reverse transcribed using Primer p(dT)15 for cDNA synthesis (Roche Diagnostic, ref 10814270001, Meylan, France) and 200 U of SuperScript II (Invitrogen, ref: 18064–014, Cergy Pontoise, France) according to the manufacturer’s instructions. The resulting tissue cDNA samples were completed to 50 μl and diluted 1/50 before PCR. The assay for each gene consisted of a pool of three independent replicates per tissue.

QPCR for compartment specific genes (17 genes) was performed using 3 μl of the final dilution (~1.5 ng LCM-derived cDNA samples (31 LCM-derived samples) and 2.4 ng tissue cDNA samples (12 tissue + fetal ovary) using SYBR green fluorescence detection during amplification on an ABI Prism 7900 Sequence Detection System 2.1 (Applied Biosystems®) as previously described [12]. The efficiency of real-time PCR amplification was calculated for each primer pair using five serial dilution points from an sheep fetal ovary cDNA sample (1:6 (~16 ng cDNA/point); 1:3; 1:3; 1:2; 1:2).

The expression of genes involved in canonical pathways (28 genes) was analyzed using 96.96 Dynamic Array™ IFCs and the BioMark™ HD System from Fluidigm. Two specific target amplifications (STA) were performed on the 36 cDNA samples (31 LCM-derived aRNA samples, 3 multi-tissue aRNA samples, a calibrator sample (pool of all the LCM-derived aRNA samples) and a pool of cDNA from total fetal ovary RNA (for the calculation of PCR amplification efficiency) in 96-well PCR plates. A mix was prepared containing 132 μL 2X TaqMan® PreAmp Master Mix plus (ref 4391128, Applied Biosystem) 26.4 μL 10X Preamplification Primer Mix (500 nM each primer (for 60 genes + 2 reference genes (*β-actin* and *TMED4*)), and 3.75 μL of this mix was added to each cDNA sample from aRNA (2 μl: 1 ng cDNA) and fetal ovary cDNA pool (2 μl =11 ng). Following a brief vortex and centrifugation, the plate was transferred to a thermal cycler and subjected to the following thermal protocol: 95°C for 10 min; 14/17 cycles of (95°C for 15 s; 60°C for 4 min); 4°C hold. *RLP19* gene primers were not included in the STA Primer Mix. The resulting STA cDNA samples were then treated with Exonuclease I to digest the primers. A mix was prepared containing 24 μL 20 units/μL Exonuclease I (M02935, NEB), 12 μL 10 x Exonuclease I Reaction Buffer and 84 μL H_2_O. Two μL of this mix was added to 5 μl of each STA cDNA sample. Following a brief vortex and centrifugation, the plate was transferred to a thermal cycler and subjected to the following thermal protocol: 37°C for 30 min; 80°C for 15 min; 4°C hold. 3.5 μl of each STA cDNA sample were diluted by adding 9 μL buffer consisting of 10 mM Tris–HCl, pH 8.0; 0.1 mM EDTA (1/5 dilution) and stored at −20°C.

For the determination of the PCR amplification efficiency specific to each gene, the STA cDNA sample of the fetal ovary cDNA pool (11 ng cDNA) was serial diluted (1, 1:3; 1:3; 1:2; 1:2).

In order to load the chip, a sample pre-mix and primer pre-mix were prepared. The sample pre-mix consisted of 440 μL 2X Taqman Gene Expression Master Mix (ref 4369510, Applied Biosystem), 44 μL 20X DNA Binding Dye Sample Loading Reagent (ref 100–0388, Applied Biosystem), 44 μl 20X EvaGreen (ref BI1790, Interchim) and 132 μL 1X TE. A 6 μL aliquot of this mix was dispensed to each well of a 96-well assay plate. A 2 μL aliquot of diluted STA cDNA samples (14 cycle STA cDNA samples and 17 cycle STA cDNA samples) was added to each well and the assay plate was briefly vortexed and centrifuged. The primer pre-mix consisted of 440 μl of 2X assay loading reagent and 110 μl of 1X TE and 6 μl of this mix was dispensed to each well of a 96-well primer plate. Aliquots (2 μl, 20X) of individual primer assay were added to each well and the primer plate was briefly vortexed and centrifuged. Following priming of the IFC in the IFC Controller HX, 5 μL from each well of the 96-well assay plate (cDNA sample + reagent mix) were dispensed to each sample inlet of 96.96 IFC and 5 μL of each well of the 96-well primer plate were dispensed to each detector inlet of the 96.96 IFC. After loading, the chip was transferred to the BioMark HD and PCR was performed using the thermal protocol: UNG and Hot Start at 50°C for 120 s; 95°C for 600 s, PCR Cycles: 35 cycles of (95°C for 15 s; 60°C for 60 s) followed by a melting phase. Data was analyzed using Fluidigm Digital PCR Analysis software using the Linear (Derivative) Baseline Correction Method, the User (Global) Ct Threshold Method with the threshold set at 0.01, and a Ct Range of 12 to 25 cycles.

After determination of the threshold cycle (Ct) for each LCM-derived aRNA sample, the PFAFFL method was used to calculate the relative expression of each gene [73]. The relative expression was normalized by the corresponding geometric average of three reference genes using geNorm v3.4 [74]: *β-actin, TMED4* and *RPL19* genes, which were respectively slightly, moderately and highly expressed and not regulated during follicle development or between follicular compartments.

The significance of compartment-specific and biomarkers differential gene expression was evaluated using Student’s test after fourth square transformation.

The significance of the differential expression of genes involved in canonical pathways was tested using the one-way ANOVA model in the R statistical software system (the Comprehensive R Archive National, http://www.cran.r-project.org) after fourth square transformation of the data. For each gene, an ANOVA model was fitted, with the 2 factor stage (4 levels) and compartment (2 levels) with interactions. A backward variable selection procedure was applied as previously described [12].

### Data access

All the raw RNA-seq data have been deposited in [EMBL-EBI ArrayExpress http://www.ebi.ac.uk/arrayexpress/ under accession number E-MTAB-1587].

## Competing interests

The authors declare that they have no competing interests.

## Authors’ contributions

AB conceived and designed the study, performed the LCM experiments, analyzed the RNA-seq data, carried out the RT-PCR and statistical analyses, and was responsible for much of the writing. OB, NM and JS performed RNA sequencing. CC and SF carried out the RNA-seq bioinformatics analysis. FB and FW helped obtain embedded ovary and staining sections. PM and BMP contributed to the conception and design of the study, and were involved in the drafting and revising the manuscript. All authors have read and approved the final version of the manuscript.

## Supplementary Material

Additional file 1 **Supplemental Figures.** This section includes 9 Supplemental Figures that illustrate the RNAseq design, bioinformatics processes, validation, and signaling pathways. Click here for file

Additional file 2 **Supplemental Results and Discussion.** This section provides a detailed description of LCM, RNA-seq, expression level preservation and reproducibility results followed by a comparative study of gene expression in 5 previous studies. Click here for file

Additional file 3 Differentially expressed genes (DEG) between compartments. Click here for file

Additional file 4 **Gene expression of known genes.** *: pval < 0.05; **: pval <0.01; ***: pval < 0.005. Click here for file

Additional file 5 Compartment specific gene analysis. Click here for file

Additional file 6 Oocyte functional enrichment. Click here for file

Additional file 7 CC functional enrichment. Click here for file

Additional file 8 **Transcriptional regulators.** Ingenuity identified the cascade of upstream transcriptional regulators that explain the observed changes in gene expression in our data set. Pval is based on a significant overlap between genes in our data set and known target regulated and transcriptional regulators. The activator z-score infers the activation state to the transcriptional regulator. Without identified bias, this z-score can always be used as an independent test to select downstream regulators. Bias means activation z-score and pval must be used to select downstream regulators. The fold change column lists FC in the data set: fold change =1 corresponds to a differential expression between compartments (>2); fold change ≠ 1 refers to the differential fold change during follicular development. Click here for file

Additional file 9 Upstream regulator analysis. Click here for file

Additional file 10 Primer sequences for real-time PCR. Click here for file
