# Supplementary material for: An overview of gene expression dynamics during early ovarian folliculogenesis: specificity of follicular compartments and bi-directional dialog
Source: BMC Genomics. 2013 Dec 19;14:904. doi: 10.1186/1471-2164-14-904 (PMC3890531; doi:10.1186/1471-2164-14-904)
Supplement: Additional file 1 — Supplemental Figures. This section includes 9 Supplemental Figures that illustrate the RNAseq design, bioinformatics processes, validation, and signaling pathways. [file 1471-2164-14-904-S1.docx]

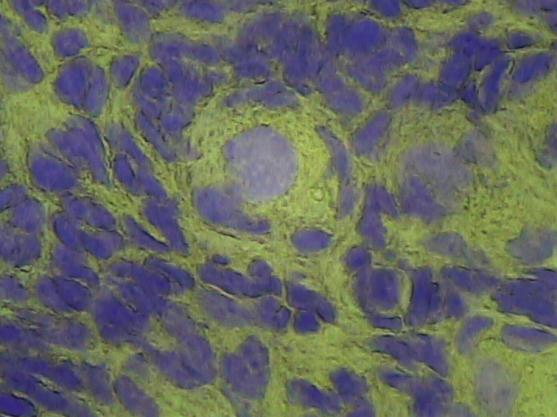

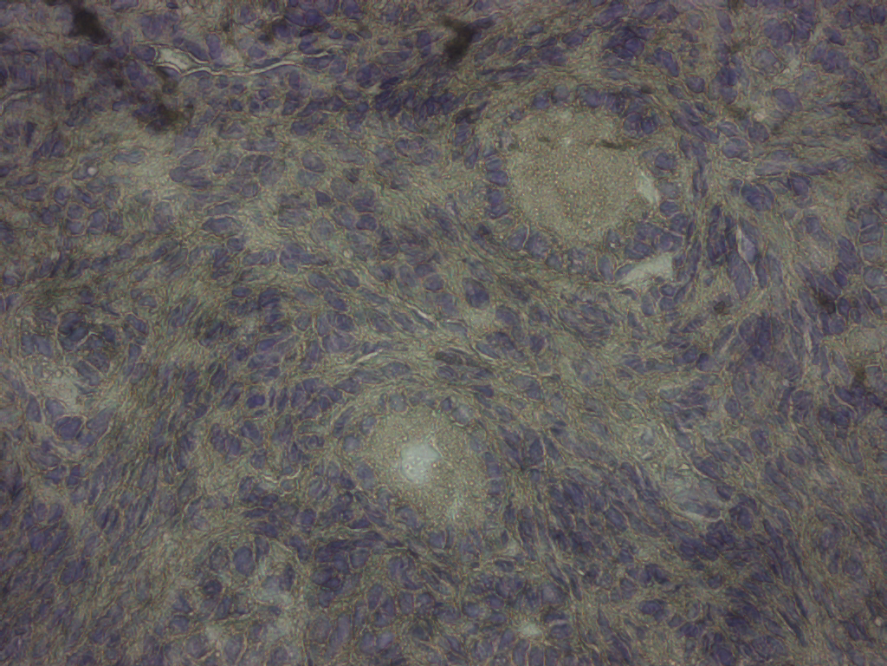

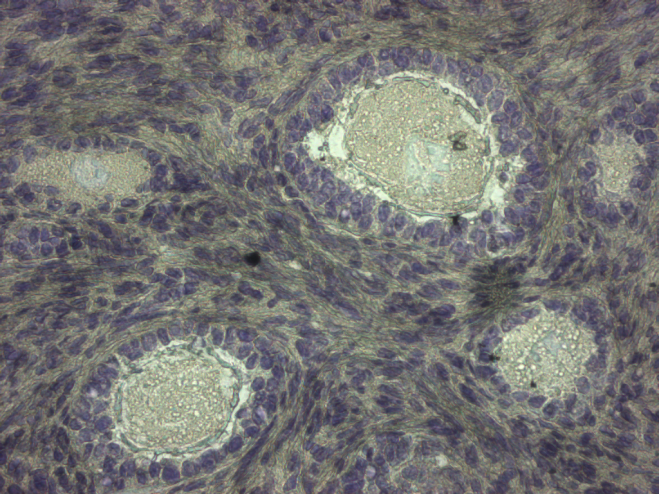

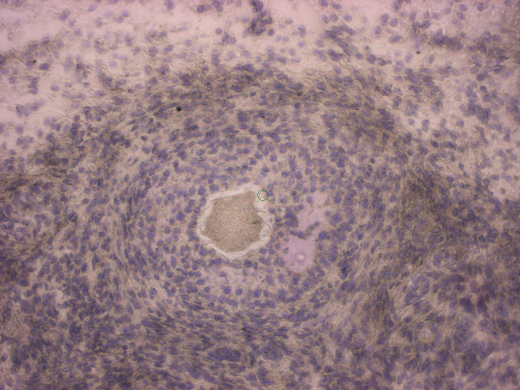


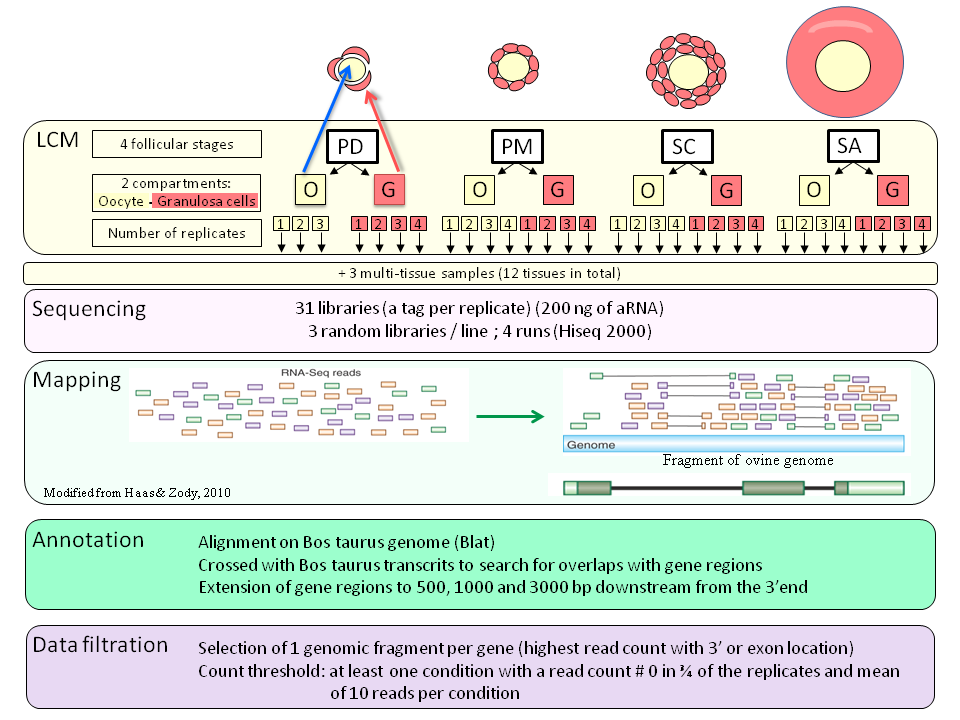


**Figure S1: Experimental design**


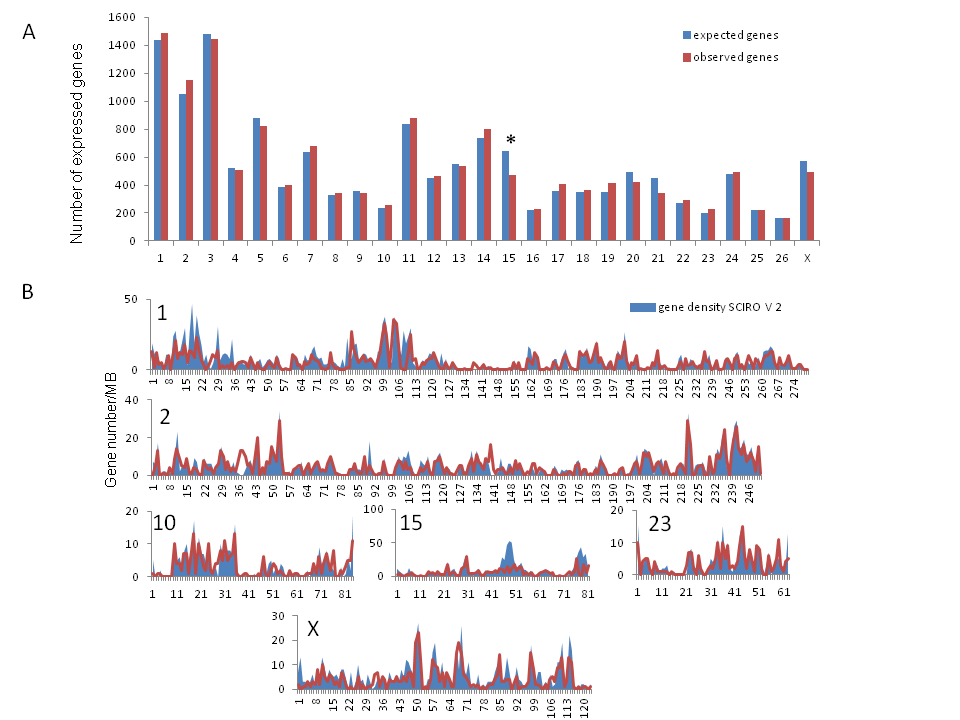


**Figure S2: Genome-wide distribution of expressed genes**

1. Number of expected vs. observed expressed genes during early folliculogenesis on chromosomes based on the sheep genome Oarv2.0 build. The genes of chromosome 15 are significantly less implicated in the follicular process (Fisher’s exact test P-value <5%).

Expected genes corresponded to 0.7865 (total expressed genes/total annotated genes (OarV2.0: 18656) X the number of annotated genes per chromosome (from OarV2.0).

1. Distribution of expressed genes on chromosomes 1, 2, 10, 15, 23, X.

The number of genes derived from OarV2.0 data are in blue. The number of expressed genes is in red.

Y axis: Number of genes in the 1MB interval

X axis: Distribution along the chromosome using 1MB interval.


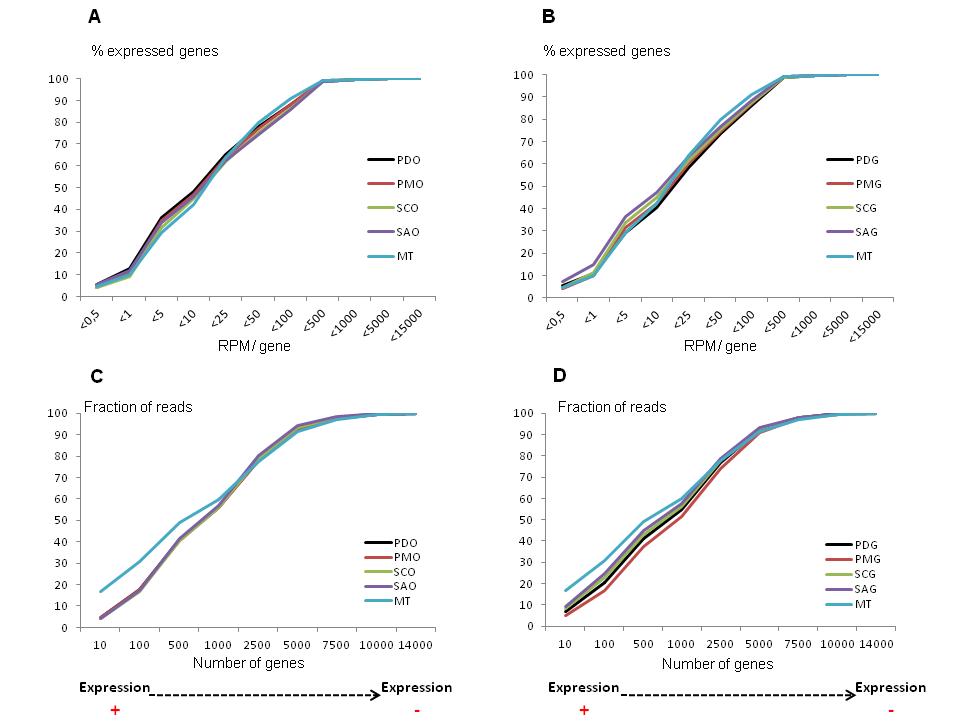


**Figure S3: transcriptome overview**

A-B) A dynamic range of gene expression:

X axis: cumulative distribution of the expression level per gene (RPM/gene: read average /gene normalized by the total number of mapped reads per sample (expressed per million of mapped reads))

Y axis: cumulative percentage of expressed genes

A: Gene expression in oocytes; B: Gene expression in granulosa cells

C-D) Complexity of transcriptomes

-C: oocyte samples; D: Granulosa cell samples

Fraction of reads derived from the most highly expressed genes in the different conditions:

For example, the 10 most expressed genes in oocyte samples (C) aggregated an average of 4.4% of the reads.

X axis: Cumulative number of genes ranked from highest to lowest expressed genes

Y axis: Cumulative fraction of reads used.


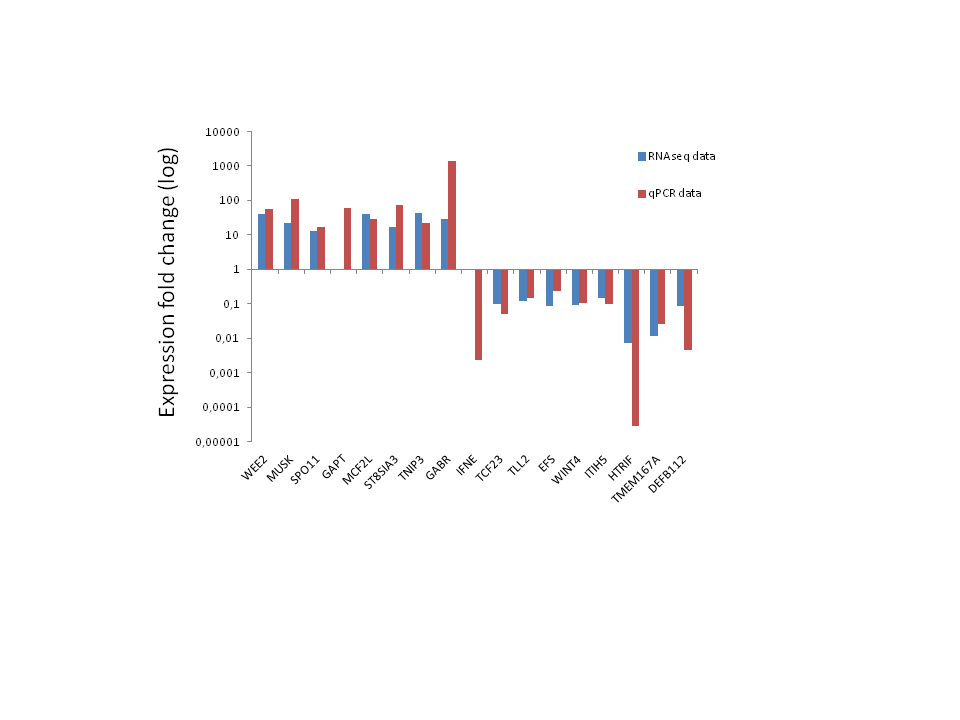


**Figure S4: RNA-seq and qPCR data comparison**

R=0.92

**
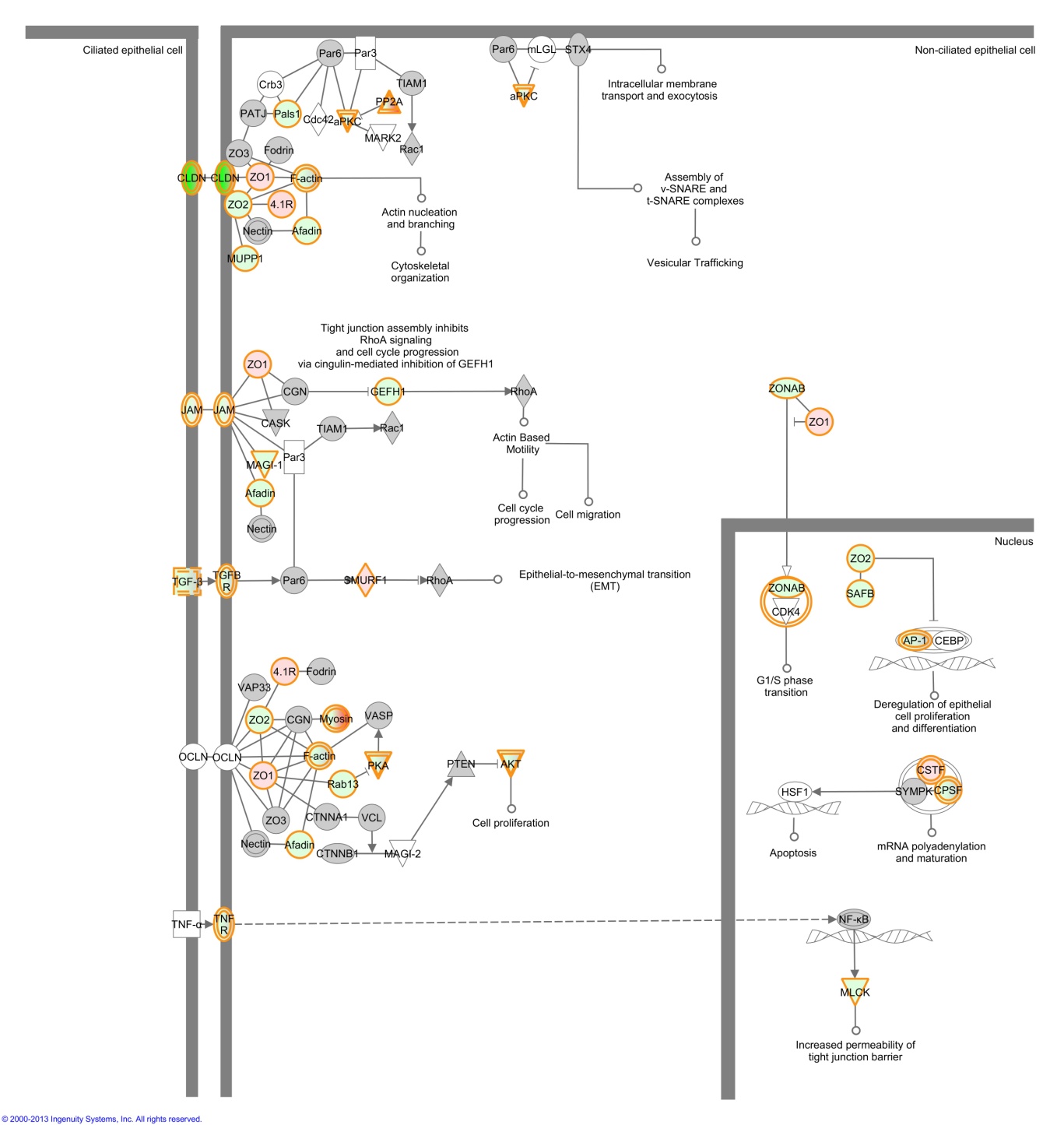
**

**Figure S5: Tight junction signaling pathway**


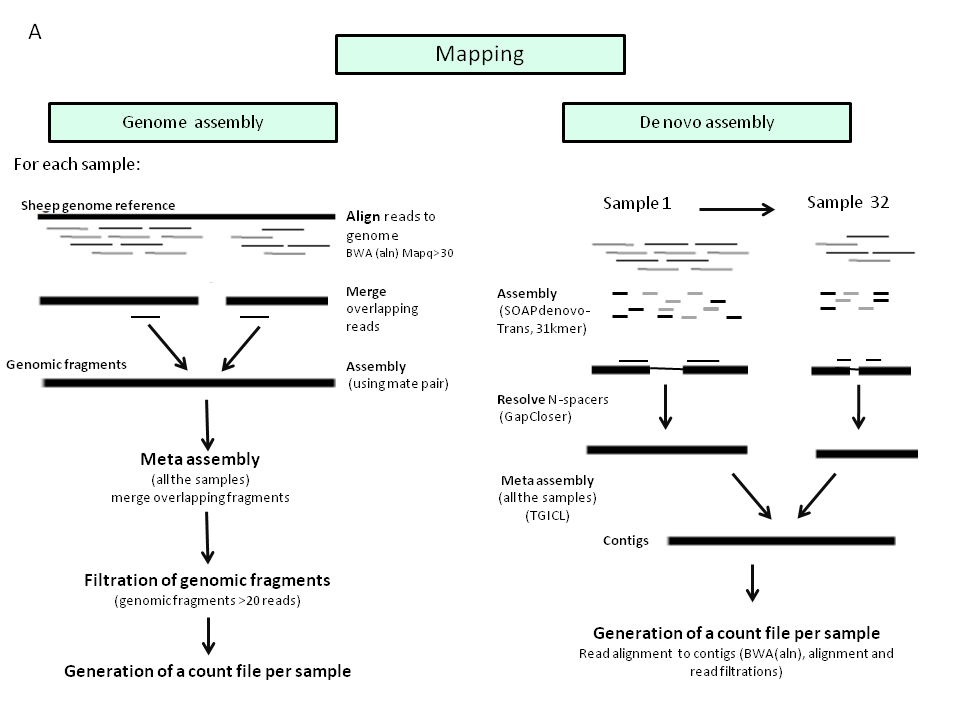


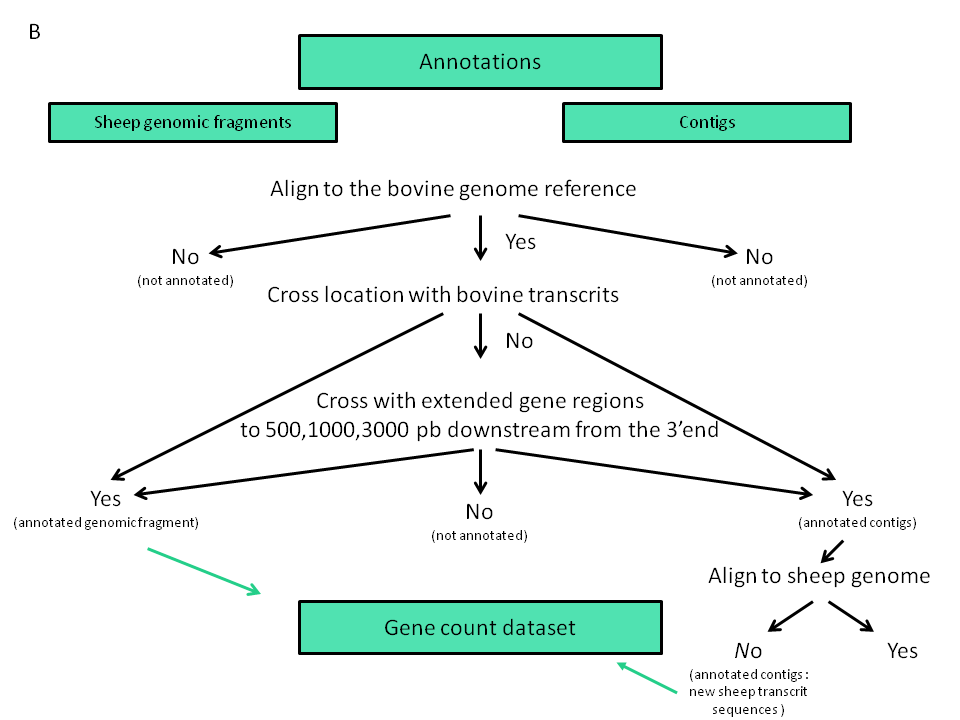


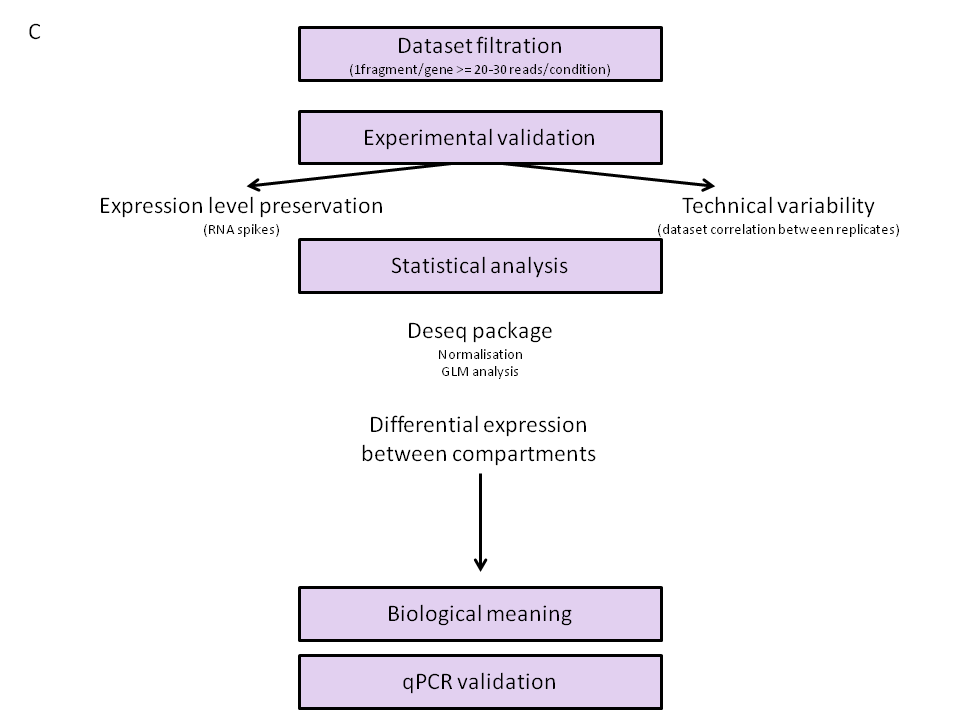


**Figure S6: workflow**

A: Bioinformatic process:

Genome assembly. After mapping to sheep genome, the overlapping reads were merged and the fragments assembled from each sample. Then, all samples were grouped and merged to produce a collection of genomic fragments. Last, the number of reads per genomic fragment was computed.

*De novo* assembly. Reads were assembled for each sample to generate contigs. All contig files were thereafter concatenated and a meta-assembly was performed. All contigs and singlets highlighted were used to create a reference and to generate a counting file for each sample.

B: Annotation process.

Genomic fragments and *de novo* contigs were aligned with the *Bos taurus* UMD3.1 genome using Blat. Iteratively, for fragments or contigs not linked to a described gene, a search was made for overlaps with downstream gene regions extended by 500bp, 1kb and 3kb. All the non-annotated genomic fragments and contigs (not mapped to the bovine transcript) were discarded from the data set. Final data set corresponds to the annotated genomic fragments.

Last, the annotated contigs (from the *de novo* assembly) were mapped to the sheep genome and added to the final data set if they did not map to sheep genome (unknown sequences in the public sheep genome database).

C: Statistical process.

The genomic data set was filtered to conserve only the best genomic fragment for each gene. After experimental validation, the normalization and significance of differential gene expression were determined using the DESeq package. Last, IPA was used to examine functional meaning.


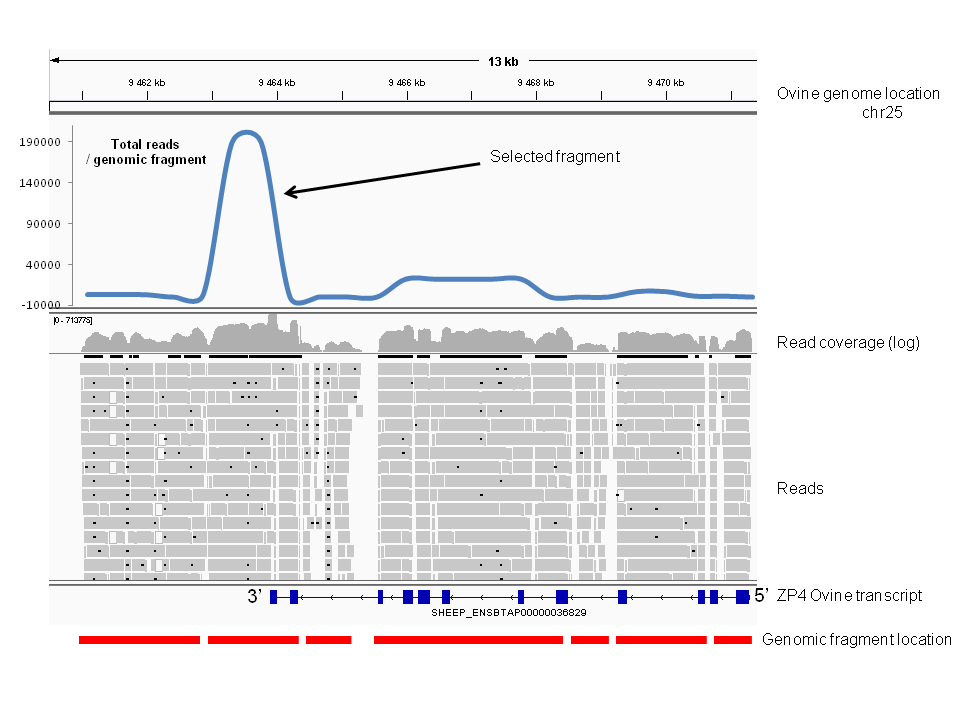


**Figure S7: Example of distribution of reads in an annotated region (ZP4 gene)**

Graphs represent from top to bottom:

- The sheep genomic location (OAR25),

- The number of total reads for each genomic fragment (y axis) identified in the dataset (blue curve),

- Read coverage (log) and read alignment visualized by IGV software,

- Location of the ZP4 gene exons (in blue).

- Location of the genomic fragments identified by the bioinformatic workflow (red line).

The non-uniformity of the sequence coverage by the reads is clearly visible with the maximum close to the 3’ UTR of the gene then a decrease towards the 5’.


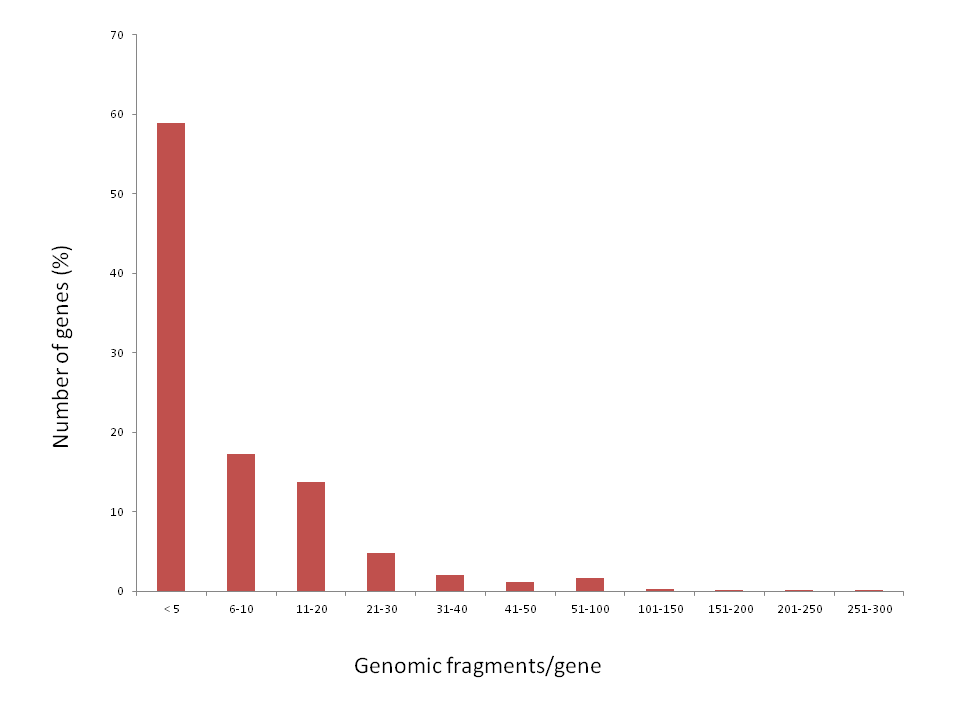


**Figure S8: Number of genomic fragments per gene**

**
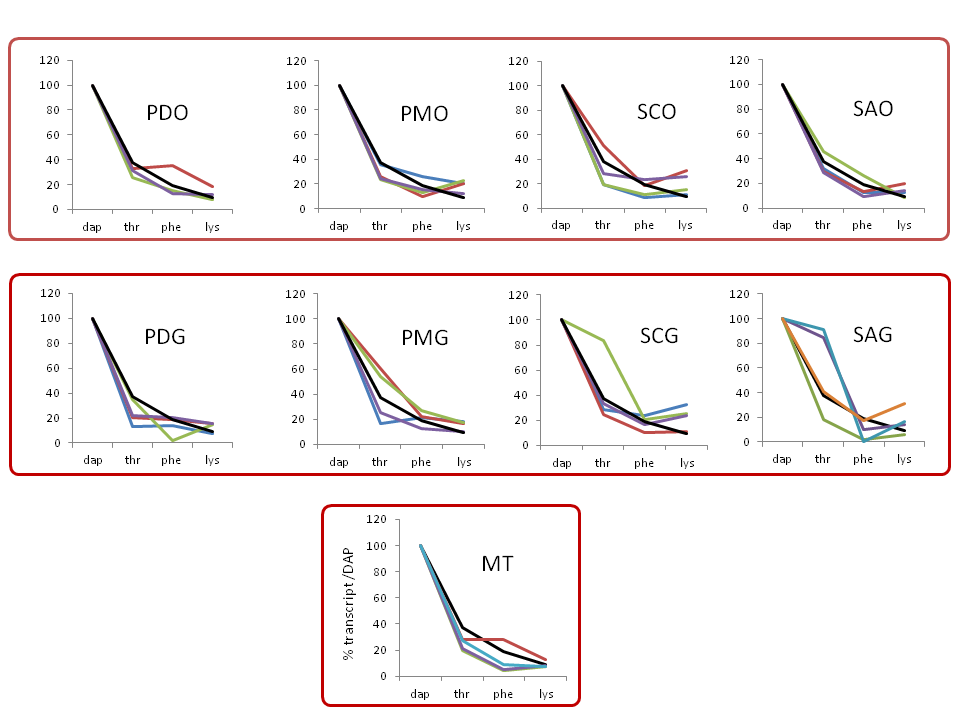
**

**Figure S9: Level of gene expression preservation**

For each condition (PDO, PMO, SCO, SAO, PDG, PMG, SCG, SAG, MT), each curve represents the percentage of measured transcripts compared to the DAP transcript.

The Y axis corresponds to the % of each transcript compared to the DAP transcript.

The black curve represents the theoretical profile (deduced from Affymetrix RNA amounts). The other colors represent the different replicates.

The profile of all the samples is similar to the theoretical profile with a correlation >0.94 except for SCG3 (0.86), SAG2 (0.8) and SAG3 (0.84).
