# Supplementary material for: An overview of gene expression dynamics during early ovarian folliculogenesis: specificity of follicular compartments and bi-directional dialog
Source: BMC Genomics. 2013 Dec 19;14:904. doi: 10.1186/1471-2164-14-904 (PMC3890531; doi:10.1186/1471-2164-14-904)
Supplement: Additional file 2 — Supplemental Results and Discussion. This section provides a detailed description of LCM, RNA-seq, expression level preservation and reproducibility results followed by a comparative study of gene expression in 5 previous studies. [file 1471-2164-14-904-S2.docx]

# Supplemental results

## Generation of compartment-specific samples using laser microdissection

To better understand transcriptome dynamics during early ovarian follicular development and molecular cross-talk between oocyte and granulosa cells (GCs), we modified and optimized the Laser Capture Microdissection (LCM) protocol [1]. We combined this technology with high throughput sequencing (RNA-sequencing) to characterize the whole transcriptome. First, using LCM, GCs and oocytes were captured separately at each stage of follicle development: primordial (PD), primary (PM), secondary (SC) follicles and the small antral stage (SA). Three/four biological replicates were obtained per condition. Due to the limited amount of total RNA generated with this procedure, LCM-RNA samples were subjected to two rounds of linear RNA amplification to obtain the amount of RNA required for Illumina cDNA library generation and QPCR validation. The experimental protocol is illustrated in Supplemental Figure S1.

## Generation of GCs and oocyte transcriptomes using RNA-sequencing

Three cDNA libraries per lane were sequenced using a Hiseq 2000 (Illumina) with a paired-end protocol. In addition, three multi-tissue-RNA samples were amplified and sequenced to highlight compartment specific transcripts (Supplemental Figure S1). We obtained around 2.647 billion 100 bp reads with an average of 73.7 million per LCM-derived amplified-RNA sample (LCM-aRNA).

Because of the animal model (sheep), two assembly strategies (genome assembly and *de novo* assembly) were evaluated to maximize transcript identification (Supplemental Figure S6A). The genome strategy produced 381 600 genomic fragments. The *de novo* assembly produced 91 378 contigs and 185 845 singlets.

The genome strategy identified 10% more genes than the *de novo* transcriptome strategy and was consequently used for further analysis. The result of the bioinformatics processing is summarized in Figure 1.

Processing produced a collection of 382 933 fragments (381 600 genomic fragments and 1 333 *de novo* contigs (genes from *de novo* strategy where the mRNA sequence was unknown in the public sheep genome or absent from the genome strategy dataset) that aggregated 47.5% of the LCM-aRNA reads. Last, the annotation strategy based on the bovine genomic sequence homology and annotation search was extended to downstream regions of the genes (500 bp, 1 kb and 3 kb, Supplemental Figure S6B) improved the read annotation by 8% and assigned 73% of the mapped reads. This strategy revealed a longer 3’ untranslated region than available in the EMBL sequence database for at least 3 186 genes (from the final data set). A total of 221 716 genomic fragments remain unannotated.

The result of the assembly and annotation processes showed that the read distributed along the genes clustered mostly towards the 3’ UTR ends and is illustrated with ZP4 gene in Supplemental Figure S7. This 3’ bias was expected and reflects the RNA amplification that follows LCM [2, 3]. This bias increased the heterogeneity of expression along the genes. A total of 86.8% of the annotated reads were located in stop codon or 3’UTR regions, whereas only 5.5% were located in exons, 1.2% in start codon or 5’UTR regions, and 6.5% in introns (Figure 1). As reported by Ameur and Teichert [3, 4], the presence of intronic RNA might represent incompletely processed transcripts or alternative splicing events. In addition, we observed that a gene was represented by a median number of eight fragments (Supplemental Figures S7-8). Consequently, to quantify gene expression, the final dataset conserved a single fragment per gene that located closest to the 3’UTR region with the highest number of reads and aggregated 89.4% of the annotated LCM-aRNA reads (86.8% were located in 3’ UTR regions and 2.6% were located in exons). For each sample, supplemental Table 1 (in Supplemental Results and Discussion) summarizes the number of reads, fragments and genes identified during the bioinformatic workflow.

The level of expression throughout the experiment (amplification, RNA-seq) was examined using a set of 4 *B*. *subtilis* transcripts (Supplemental Figure S6C). Supplemental Figure S9 shows that the expression profile of these transcripts was similar to the theoretical expression profile (derived from the Affymetrix amount) for all the samples (correlation >0.8). As previously described [5, 6], the two rounds of amplification and RNA-seq processes resulted in no significant distortion of the transcript population. Finally, to evaluate the reproducibility of the RNA-seq measure, PDG4 was sequenced twice (PDG4 and PDG4B). The two technical replicate files showed a good correlation (r=0.99) and indicated that the transcript abundance measurement was reproducible.

# Supplemental Discussion

## Comparative studies of gene expression

This RNA-seq study documented the global expression of 15 349 genes in ovarian follicles during early follicular development in sheep. Using this technology, we estimated a larger number of genes expressed in oocytes (14 172 genes expressed in ¾ of replicates) than other microarray studies performed on mouse and human during early follicular development. Pan et al. detected around 9 330 unigenes in PD, PM, SC and SA mouse oocytes [7] and Markholt et al. found a total of 6 301 unique genes expressed in PD/PM human oocytes [8].

Compared to our preliminary study [1], we found that RNA-seq technology was better and more sensitive for the study of basal folliculogenesis in sheep. In practice, microarray supports are often poorly annotated, poorly oriented and incomplete for non-model species and do not enable the study of complete processes like folliculogenesis [9]. On one hand, the bovine Affymetrix chip included 24 024 probes of which only 64% are annotated, corresponding to 12 404 unique genes. In addition, a great number of known ovarian genes are not present on bovine Affymetrix chip (37% of the oocyte genes and 47% of the GC genes already identified in four previous studies [1]). On the other hand, our RNA-seq experiment identified the expression of 2.5 times more genes (14 561 genes in PD, PM and SC samples compared to 5 909 genes in the Affymetrix experiment). This significant difference between the two technologies can be attributed to more exhaustive detection combined with better detection by RNA-seq of weakly expressed genes. Indeed, we identified a large number of genes with a lower expression (the median expression was 140 RPM in a scale that ranged from 0.2 to 1 000 RPM). Finally, RNA-seq detected an additional 20% of known mouse genes compared to the bovine Affymetrix support. An increase of 22% in the number of genes detected by RNA-seq versus Affymetrix chip was also mentioned with respect to human colon cancer by Xu *et* *al.* [10]. Sixty-six percent of the specifically expressed genes reported in our preliminary sheep study using the bovine Affymetrix chip [1] were detected by RNA-seq but only 23% of them were confirmed as differentially expressed by DESeq (Supplemental Table 2: in supplemental Results and Discussion). Indeed, RNA-seq statistical analysis described and accounted for the marked variation in biological replicates (3-4 biological replicates) and produced a more robust statistic (pval<0.5%) than previous microarray data (without replicate).

Finally, our RNA-seq data recovered between 44% and 76% of the genes previously described in studies of mouse oocytes/ Paillisson *et al*. [11], Pan *et al* [7] and Gallardo *et al*. [12], Arraztoa *et al*. [13] (Supplemental Table 2: from supplemental Results and Discussion).

# Supplemental Tables

### Table 1 - Summary of bioinformatics data processing

Summary of the results of data set processing in terms of the number of reads (columns 2-5) and genomic fragments (columns 6-8) for:

1. Mapping against the sheep genome sequence and the bovine genome sequence (for annotated *de novo* contigs without sheep sequences)(columns 3 and 6),
2. The annotation using the bovine genome reference (columns 4 and 7)
3. The filtration process (columns 5 and 8)(see methods)

Each independent biological replicate is denoted 1, 2, 3, or 4.

* corresponds to the number of expressed genes (see Methods: a single genomic fragment/ annotation)

| Sample | Number of reads | Number of mapped reads | Number of annotated reads | Number of post-trimmed reads | Number of fragments | Number of annotated fragments | Number of post-trimmed annotated fragments* |
| --- | --- | --- | --- | --- | --- | --- | --- |
| PDO1 | 59107022 | 29491038 | 21909066 | 19631107 | 136856 | 66644 | 14239 |
| PDO2 | 78712828 | 36565964 | 26131630 | 22729122 | 106849 | 52346 | 13455 |
| PDO3 | 52507048 | 25887008 | 18696000 | 16543170 | 83224 | 41212 | 12545 |
| PMO1 | 66533942 | 31717742 | 23153342 | 20776619 | 100956 | 50312 | 13417 |
| PMO2 | 54853348 | 28382894 | 20326266 | 17842928 | 100424 | 48642 | 13237 |
| PMO3 | 74817756 | 36443126 | 25827283 | 22727998 | 82386 | 39525 | 12267 |
| PMO4 | 67907894 | 34265311 | 24841668 | 21911423 | 167705 | 77813 | 14504 |
| SCO1 | 28458350 | 13823263 | 9943772 | 8772385 | 73667 | 37139 | 12227 |
| SCO2 | 76037636 | 34143054 | 23995757 | 20995166 | 56313 | 29439 | 11590 |
| SCO3 | 134799296 | 67472656 | 48449300 | 43076217 | 91600 | 45448 | 13181 |
| SCO4 | 82581444 | 40182001 | 28964036 | 25669933 | 103392 | 50055 | 13261 |
| SAO1 | 104296372 | 47013302 | 33675297 | 29846215 | 63058 | 33146 | 12037 |
| SAO2 | 76471372 | 39081300 | 29148055 | 25856211 | 71998 | 38993 | 12038 |
| SAO3 | 48871602 | 26189650 | 19199688 | 17002822 | 97704 | 48588 | 12919 |
| SAO4 | 86505876 | 43520726 | 31963871 | 28382605 | 105008 | 52207 | 12984 |
| PDG1 | 86604614 | 37224960 | 26966837 | 23901793 | 42206 | 24291 | 11131 |
| PDG2 | 54018012 | 25413078 | 19392375 | 17696441 | 113951 | 59895 | 13841 |
| PDG3 | 68940288 | 30094125 | 22381382 | 20121546 | 116789 | 61000 | 14153 |
| PDG4 | 115879260 | 51665660 | 38470899 | 35310535 | 56959 | 30249 | 11905 |
| PDG4bis | 107046614 | 56024584 | 41736494 | 38332627 | 35454 | 21782 | 10890 |
| PMG1 | 62812266 | 29252010 | 21649829 | 19295340 | 90234 | 47001 | 13230 |
| PMG2 | 85018548 | 41612883 | 30941975 | 27962486 | 122106 | 63168 | 14126 |
| PMG3 | 63606594 | 29225551 | 21444332 | 19192578 | 73164 | 39176 | 12762 |
| PMG4 | 86011686 | 41612635 | 30220703 | 26903780 | 118362 | 57917 | 13789 |
| SCG1 | 53898266 | 26460705 | 19580775 | 17755101 | 113648 | 58361 | 13784 |
| SCG2 | 52965602 | 23645969 | 16876449 | 15337656 | 58372 | 33977 | 12293 |
| SCG3 | 80019298 | 38465753 | 28445158 | 25718671 | 114032 | 59356 | 13778 |
| SCG4 | 77829738 | 32695128 | 23602913 | 21414045 | 78404 | 43141 | 12848 |
| SAG1 | 59971074 | 25976393 | 18723373 | 16636318 | 124343 | 68239 | 13967 |
| SAG2 | 61153832 | 30256762 | 22904620 | 20571566 | 171387 | 86781 | 14614 |
| SAG3 | 66867882 | 31460220 | 23000486 | 20452438 | 99503 | 56172 | 12912 |
| SAG4 | 82098424 | 35892383 | 26382440 | 24039685 | 105829 | 57793 | 13268 |
| MT1 | 101386238 | 45749122 | 33890909 | 29529526 | 75286 | 43735 | 12748 |
| MT2 | 95179366 | 41085315 | 29208160 | 25055305 | 69103 | 40702 | 12761 |
| MT3 | 93123920 | 41470348 | 29655324 | 25178294 | 74152 | 43671 | 12850 |
| total | 2646893308 | 1249462619 | 911700464 | 812169652 | 382933 | 161211 | 15349 |

## Table 2 - Comparative studies of gene expression

Literature data:

1- Bonnet: Transcriptome profiling of sheep granulosa cells and oocytes during early follicular development obtained by Laser Capture Microdissection (Affymetrix bovine chip)

1. Dadé: Differentially expressed genes in mouse oocytes compared to other tissues.

The selection was performed by *in silico* differential display between three mouse oocyte cDNA libraries and 13 selected tissues cDNA libraries.

1. Gallardo: set of ovarian factors from mouse Foxo3 ovaries.

Gene classes were obtained by comparative profiling from mouse Affymetrix data sets including ovary RNA extracted at four time points spanning follicle assembly and early growth, and 14 somatic tissues containing LCM primary oocytes and LCM somatic cells.

1. Pan: The overall change in oocyte gene expression was characterized using Pd, Pm, Sec, SA and antral mouse follicles.

- Mouse oocyte differentially expressed genes between primordial and primary follicular stages.

1. Arraztoa: Primate oocyte-enriched transcripts between the microdissected primordial stage and placenta RNA (control).

| **References** | **Species** | **Compartment** | **Data** | **DEG number** | RNAseq Data | | |
| --- | --- | --- | --- | --- | --- | --- | --- |
|  |  |  |  |  | **No. of genes detected** | **O/GCs**  **No. of differential genes** | **GCs/O**  **No. of differential genes** |
| **Experiment in present study** | Sheep | O/GCs |  | 5130 |  | 2297 | 2832 |
| **Bonnet [1]** | Sheep | Oocyte | Over-expressed in oocyte | 759 | 505 | 102 | 63 |
|  |  | GCs | Over-expressed in GCs | 1050 | 690 | 66 | 175 |
| **Paillisson [7]** | Mouse | Oocyte | Enriched by In silico DD | 104 | 79 | 26 | 11 |
| **Gallardo [9]** | Mouse | Oocyte | Class IA-follicle assembly/meiosis | 32 | 14 | 6 | 2 |
|  |  |  | Class IC-oocyte-specific, early maturation only | 14 | 8 | 5 | 1 |
|  |  |  | Class IB-oocyte-specific, early, and late maturation | 66 | 28 | 17 | 1 |
|  |  |  | Class III-unfertilized egg | 84 | 46 | 11 | 12 |
|  |  | Somatic cells | Class ID-follicle growth, somatic | 24 | 21 | 0 | 13 |
| **Pan [8]** | Mouse |  | Over-expressed in oocyte | 2578 | 1908 | 428 | 227 |
|  |  | Oocyte PD/PM | Increase | 197 | 125 | 18 | 6 |
|  |  |  | Decrease | 213 | 121 | 13 | 6 |
| **Arraztoa [10]** | Monkey | Oocyte PD | Enriched/placenta | 79 | 36 | 4 | 9 |

# References

1. Bonnet A, Bevilacqua C, Benne F, Bodin L, Cotinot C, Liaubet L, Sancristobal M, Sarry J, Terenina E, Martin P *et al*: **Transcriptome profiling of sheep granulosa cells and oocytes during early follicular development obtained by laser capture microdissection**. *BMC Genomics* 2011, **12**:417.

2. Schmid MW, Schmidt A, Klostermeier UC, Barann M, Rosenstiel P, Grossniklaus U: **A powerful method for transcriptional profiling of specific cell types in eukaryotes: laser-assisted microdissection and RNA sequencing**. *PloS one* 2012, **7**(1):e29685-e29685.

3. Teichert I, Wolff G, Kueck U, Nowrousian M: **Combining laser microdissection and RNA-seq to chart the transcriptional landscape of fungal development**. *Bmc Genomics* 2012, **13**.

4. Ameur A, Zaghlool A, Halvardson J, Wetterbom A, Gyllensten U, Cavelier L, Feuk L: **Total RNA sequencing reveals nascent transcription and widespread co-transcriptional splicing in the human brain**. *Nature Structural & Molecular Biology* 2011, **18**(12):1435-U1157.

5. Nakazono M, Qiu F, Borsuk LA, Schnable PS: **Laser-capture microdissection, a tool for the global analysis of gene expression in specific plant cell types: Identification of genes expressed differentially in epidermal cells or vascular tissues of maize. (vol 15, pg 583, 2003)**. *Plant Cell* 2003, **15**(4):1049-1049.

6. Lang JE, Magbanua MJ, Scott JH, Makrigiorgos GM, Wang G, Federman S, Esserman LJ, Park JW, Haqq CM: **A comparison of RNA amplification techniques at sub-nanogram input concentration**. *BMC Genomics* 2009, **10**:326.

7. Pan H, O'Brien M J, Wigglesworth K, Eppig JJ, Schultz RM: **Transcript profiling during mouse oocyte development and the effect of gonadotropin priming and development in vitro**. *Dev Biol* 2005, **286**(2):493-506.

8. Markholt S, Grondahl ML, Ernst EH, Andersen CY, Ernst E, Lykke-Hartmann K: **Global gene analysis of oocytes from early stages in human folliculogenesis shows high expression of novel genes in reproduction**. *Molecular Human Reproduction* 2012, **18**(2):96-110.

9. Bonnet A, Dalbiès-Tran R, Sirard MA: **Opportunities and challenges in applying genomics to the study of oogenesis and folliculogenesis in farm animals**. *Reproduction* 2008, **135**(2):119-128.

10. Xu X, Zhang Y, Williams J, Antoniou E, McCombie WR, Wu S, Zhu W, Davidson NO, Denoya P, Li E: **Parallel comparison of Illumina RNA-Seq and Affymetrix microarray platforms on transcriptomic profiles generated from 5-aza-deoxy-cytidine treated HT-29 colon cancer cells and simulated datasets**. *BMC Bioinformatics* 2013, **14 Suppl 9**:S1.

11. Paillisson A, Dade S, Callebaut I, Bontoux M, Dalbies-Tran R, Vaiman D, Monget P: **Identification, characterization and metagenome analysis of oocyte-specific genes organized in clusters in the mouse genome**. *BMC Genomics* 2005, **6**(1):76.

12. Gallardo TD, John GB, Shirley L, Contreras CM, Akbay EA, Haynie JM, Ward SE, Shidler MJ, Castrillon DH: **Genomewide discovery and classification of candidate ovarian fertility genes in the mouse**. *Genetics* 2007, **177**(1):179-194.

13. Arraztoa JA, Zhou J, Marcu D, Cheng C, Bonner R, Chen M, Xiang C, Brownstein M, Maisey K, Imarai M *et al*: **Identification of genes expressed in primate primordial oocytes**. *Hum Reprod* 2005, **20**(2):476-483.
